# Centenarian hippocampus displays high levels of astrocytic metallothioneins

**DOI:** 10.1111/acel.14201

**Published:** 2024-05-20

**Authors:** Ander Saenz‐Antoñanzas, Maider Muñoz‐Culla, Piero Rigo, Leire Ruiz‐Barreiro, Manuel Moreno‐Valladares, Ainhoa Alberro, Sara Cruces‐Salguero, Marta Arroyo‐Izaga, Amaia M. Arranz, David Otaegui, François Guillemot, Ander Matheu

**Affiliations:** ^1^ Cellular Oncology Group Biodonostia Health Research Institute San Sebastian Spain; ^2^ Multiple Sclerosis Group Biodonostia Health Research Institute San Sebastian Spain; ^3^ CIBERNED, ISCIII Madrid Spain; ^4^ Department of Basic Psychological Processes and their Development University of the Basque Country (UPV/EHU) San Sebastian Spain; ^5^ Neural Stem Cell Biology Laboratory The Francis Crick Institute London UK; ^6^ Laboratory of Humanized Models of Disease, Achucarro Basque Center for Neuroscience Leioa Spain; ^7^ BIOMICs Research Group, Microfluidics & BIOMICs, Department of Pharmacy and Food Sciences, Lascaray Research Center University of the Basque Country (UPV/EHU), Bioaraba Vitoria Spain; ^8^ IKERBASQUE, Basque Foundation for Science Bilbao Spain; ^9^ CIBERFES, ISCIII Madrid Spain

**Keywords:** astrocytes, centenarians, hippocampus, metallothioneins, transcriptomic

## Abstract

The hippocampus is a brain area linked to cognition. The mechanisms that maintain cognitive activity in humans are poorly understood. Centenarians display extreme longevity which is generally accompanied by better quality of life, lower cognitive impairment, and reduced incidence of pathologies including neurodegenerative diseases. We performed transcriptomic studies in hippocampus samples from individuals of different ages (centenarians [≥97 years], old, and young) and identified a differential gene expression pattern in centenarians compared to the other two groups. In particular, several isoforms of metallothioneins (MTs) were highly expressed in centenarians. Moreover, we identified that MTs were mainly expressed in astrocytes. Functional studies in human primary astrocytes revealed that MT1 and MT3 are necessary for their homeostasis maintenance. Overall, these results indicate that the expression of MTs specifically in astrocytes is a mechanism for protection during aging.

## Graphical abstract

Higher levels of MT1 and MT3 are detected in hippocampus of very old individuals (over 90) compared with old and young individuals. MTs colocalize with astrocytic markers.

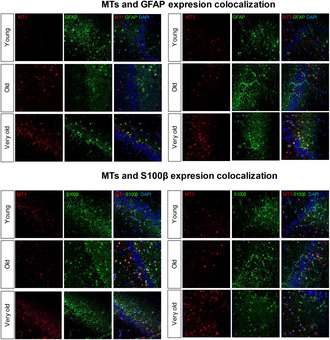

## 1 INTRODUCTION

Aging is a systemic, multifactorial, and degenerative process characterized by the decline and loss of physical and mental capacities (Lopez‐Otin et al., 2023). Although human aging affects the entire organism, brain aging is especially distinctive. The brain is probably the most structurally and functionally complex organ, which commands multiple cognitive abilities, including communication, thinking, memory, movement, and emotions. All of them suffer a functional decline with aging until they reach a point in which the individual is not able to perform the activities of daily living (Murman, 2015). Cognitive activities are regulated in part by the two neurogenic niches, the subgranular zone of the dentate gyrus (DG) in the hippocampus, that is, vital for learning and memory and the subventricular zone.

Human brain contains billions of neurons, which are surrounded by a vast number of glial cells, whose presence in the brain, compared to the neuron number, is equivalent to or even greater in some brain regions (von Bartheld et al., 2016). The main glial cells from the central nervous system (CNS) include macroglia (astrocytes, oligodendrocytes, and ependymal cells) and microglia, all of them contributing and regulating different processes such as the correct neurotransmission, neural damage repair, metabolism, blood flow to the brain, or even adult neurogenesis (Salas et al., 2020). The changes that occur in the brain with aging include alterations in the structure and function of neurons, loss of synapses, or dysfunctions in neuronal networks. Besides neurons, glial cells are also affected by aging, losing their ability to carry out their normal functions and releasing detrimental factors that can affect other cells of the brain (Blinkouskaya et al., 2021; Salas et al., 2020). In this direction, recent transcriptomic studies at the single‐cell level in mice have revealed widespread changes in specific glial cell types such as microglia and astrocytes with age and highlighted increased inflammation as a key aspect of brain aging (Allen et al., 2023; Ximerakis et al., 2019). These alterations in the hippocampus promote a decline in cognitive abilities and the onset of dementia (Bettio et al., 2017). Thus, it is vital to understand how age impacts cognitive function at cellular and molecular levels.

Previous studies of the aging human brain have shown dynamic gene expression changes that distinguish young adults from the aging population (Ham & Lee, 2020). Independent transcriptome analyses showed shifts in the expression of different glial‐specific genes and indicated that inflammatory or immune‐responsive genes are upregulated during aging in most brain regions (Lu et al., 2004; Olah et al., 2018; Soreq et al., 2017), increasing the vulnerability of the brain to cognitive aging. Moreover, a recent transcriptomic study performed in frontal cortex samples of individuals organized in two different groups according to their age (≤80 vs. ≥85 years) showed that the ≥85 years group of age was associated with a distinct transcriptome signature in the cerebral cortex and revealed a protective mechanism of aging and longevity mediated by neural circuit activity and regulated by REST transcription factor (Zullo et al., 2019).

Centenarians are a group that exhibits extreme longevity, which is commonly accompanied by better quality of life, physical independence, and cognitive function compared to older individuals dying in 70s or 80s (Evert et al., 2003; Hitt et al., 1999). Additionally, they usually exhibit medical histories with low incidence rates of age‐associated common and lethal pathologies, such as stroke, cancer, or cardiovascular disease (Ismail et al., 2016). Among the centenarians, there is the subgroup of supercentenarians that display even higher resistance to lethal diseases and are able to approximate healthspan to lifespan to the limit (Andersen et al., 2012), having unique biological characteristics by sustaining immune responses to protect them from infections and diseases (Hashimoto et al., 2019). Omic approaches in blood samples from centenarians revealed that the transcriptome and the expression pattern of noncoding RNAs are more similar to young individuals than septuagenarians (Borras et al., 2016; Serna et al., 2012). Moreover, their blood samples overexpress pluripotency‐ and stemness‐related genes (Inglés et al., 2019) show reduced expression of senescence markers and present more favorable biomarkers of metabolism, inflammation, and anemia than individuals dying earlier (Murata et al., 2024), overall supporting the idea that centenarians and very old individuals differ from the old and resemble young individuals at biological and molecular levels.

Different studies have also described that centenarians present a reduced number of cases with neurodegenerative diseases, in some cases avoid dementia, and also display lower levels of Alzheimer's disease (AD) pathology, including beta‐amyloid and ApoE4 allele (Andersen, 2020; Calvert et al., 2006; von Gunten et al., 2010). Consistent with these ideas, our recent study characterizing Basque centenarians identified that they showed better biological profiles in blood analysis, required fewer use of medical resources, and developed fewer diseases including from the nervous system compared with non‐centenarians (Cruces‐Salguero et al., 2022). Additional studies showed that incidence and pathology of AD reach a peak around 95 years of age and is lower afterward (Farfel et al., 2019; Savva et al., 2009), and the prevalence of escapers shows a valley between 70 and 90 years of lifespan and increases afterward (Sol et al., 2024). Thus, in order to understand the mechanisms associated with cognitive decline and aging, we performed transcriptomic analysis in hippocampus samples of human individuals of different ages, including young, old, and centenarians (individuals of ≥97 years).

## 2 MATERIALS AND METHODS

### 2.1 Human brain samples and publicly available data sets

For transcriptome and RT‐qPCR studies, the BIOMICs group provided the human brain samples obtained from forensic autopsy, which are part of the collection C.0000217 from Instituto Salud Carlos III Biobank register (https://biobancos.isciii.es/ListadoColecciones.aspx). The transcriptomic study was performed in coronal sections of human hippocampal samples from 16 individuals, including young individuals (*n* = 5, 27–49 years old), old (*n* = 8, 58–88 years old), and very old and centenarians (*n* = 3, 97, 99, and 100 years old). RT‐qPCR validation was completed in additional samples of 33 individuals, including young (*n* = 16, 27–45 years old), old (*n* = 13, 76–82), and very old individuals (*n* = 4, 92–96) (Auzmendi‐Iriarte et al., 2022).

Inmunofluorescence (IF) studies were completed in paraffin sections from samples obtained from Navarrabiomed Biobank (https://www.navarrabiomed.es/es/servicios/biobanco) and Donostia Hospital (Moreno‐Valladares et al., 2020) of 26 individuals, including young (*n* = 8, 26–43 years), elderly (*n* = 15, 65–84), and very old (*n* = 3, 90, 92, and 103). All individuals included in the study had no diagnosis of neurodegenerative disorders as well as no neuropathological injuries in the regions analyzed.

Additionally, single‐cell RNAseq data from the brain was analyzed from “The Human Protein Atlas” (http://www.proteinatlas.org) using the consensus data set consisting of the normalized expression (nTPM) levels and RNAseq data of progenitor and mature human astrocytes from (available at http://www.brainrnaseq.org). RNAseq data originated from the bulk human dorsolateral prefrontal cortex (*n* = 540) and purified human microglia (*n* = 10) from the same brain region were also analyzed (http://shiny.maths.usyd.edu.au/Ellis/MicrogliaPlots/). In situ hybridization data were obtained from sagittal sections of P56 male adult C57BL/6J mice from the Allen Brain Atlas data portal (https://mouse.brain‐map.org/).

### 2.2 Transcriptome analysis

Gene expression array was performed from 5 ng of RNA using ClariomTM S array (Affymetrix). Labeled RNA was hybridized to the array, washed and stained in a GeneChip Fluidics Station 450, and scanned in a GeneChip Scanner 7G (Affymetrix). Microarray data were analyzed with Transcriptome Analysis Console v4.0 (TAC). Raw data were normalized using the robust multi‐array average (RMA), and the batch effect observed was eliminated using the batch effect module of the TAC software. Then, studied groups were compared with Limma differential expression analysis to find differentially expressed genes (DEGs). Differentially expressed genes with *p* < 0.05 and fold change ≥ |2| were selected. Heatmaps and hierarchical clustering of DEG were created with heatmap.2 function from “gplots” package in R environment (version 4.3.2) running in RStudio (2023.09.1). Venn diagrams were created using Venny 2.1 (https://bioinfogp.cnb.csic.es/tools/venny/index.html). Gene Ontology (GO) analysis was performed using “clusterProfiler” package also in the R environment. On the one hand, enriched pathways based on differentially upregulated genes in centenarians against either old and/or young groups were analyzed, and the same analysis was performed independently with downregulated genes. The data discussed in this publication have been deposited in NCBI's Gene Expression Omnibus and are accessible in GSE201118.

### 2.3 Astrocytes culture

Normal Human Astrocytes (NHA, ScienCell) were cultured in adhesion in culture plates pretreated with 15 μg/mL poly‐L‐lysine. Astrocyte medium kit was employed, and cells were maintained in standard culture conditions at 37°C, 95% humidity, 21% O_2_, and under 5% CO_2_ pressure. NHA were maintained in culture, and passages were performed every 4–5 days. The expression of genes was studied at early (Lopez‐Otin et al., 2023; Murman, 2015; von Bartheld et al., 2016) and late (Andersen et al., 2012; Evert et al., 2003; Hashimoto et al., 2019; Hitt et al., 1999; Ismail et al., 2016; Olah et al., 2018; Zullo et al., 2019) passages after total RNA extraction from cell cultures.

Generation of astrocytes from human pluripotent stem cell (hiPSC) lines. hPSCs from the H9 (WA09) line (WiCell Research Institute) maintained on Matrigel in mTeSR1 (StemCell Technologies) were differentiated to neural progenitor cells (NPCs) by dual SMAD inhibition (0.1 μM LDN193189 and 10 μM SB431542) in embryoid bodies (EB) media (StemCell Technologies). Rosettes were selected at day in vitro (DIV) 14 by Rosette Selection Reagent and patterned to forebrain NPCs with EB media containing 20 ng/mL FGF2. NPCs from the H9 line and from the ax0019 line purchased from Axol Bioscience were characterized immunocytochemically using SOX2, PAX6 and Nestin. Dissociated NPCs were plated at a density of 50.000 cells/cm^2^ on matrigel‐coated plates and differentiated into astrocytes. Briefly, cells were cultured in an astrocyte medium (DMEM/F‐12 + Glutamax, N2 supplement, and B27 supplement without vitamin A) supplemented with EGF and LIF for 14 days, and subsequent maturation of astrocytes was obtained by exposure to the astrocyte medium supplemented with CNTF for other 30 days. Cells were cultured and harvested at DIV 53‐62 and DIV 104‐110.

### 2.4 Lentiviral infections

NHA were transduced with lentiviral vectors containing a plasmid with silencing sequence of *MT1* or *MT3* (Sigma‐Aldrich). *pLKO.1* puro (Addgene plasmid #8453) was used as control. Lentiviral infections were performed overnight with a multicity of infection (MOI) of 10 at 37°C and 5% CO_2_ in the astrocyte medium. After 48 h, infected cells were selected in the presence of 2 μg/mL puromycin (Sigma‐Aldrich) for 48–72 h.

### 2.5 Cell growth and senescence assays

NHA were plated in a density of 10^4^ cells in six‐well plates, and the number of cells was determined on days 1, 4, and 8. Data were represented the total number of cells per experimental condition at each time point. Senescence assay was performed using the Senescence β‐Galactosidase Staining Kit (Cell Signaling) according to the manufacturer's guidelines.

### 2.6 Cell and tissue IF

Cell IF was performed using a standard protocol. Cells were incubated with primary phospho‐histone H3 (p‐H3) (Abcam), Ki67 (Abcam), Caspase 3 (R&D Systems), GFAP (Invitrogen), MT1 (Abcam), and MT3 (Sigma) antibodies and secondary anti‐mouse and anti‐rabbit (Invitrogen) antibodies. For nuclear DNA staining, Hoechst 33342 (Sigma) was used. Pictures were taken with an Eclipse 80i microscope and processed with the NIS Elements Advances Research software (Nikon).

Tissue IF was performed in formalin‐fixed brain samples. The sections were deparaffinized and incubated with 0.5% sodium borohydride for 30 min and heated in citrate buffer at pH 6 for 20 min for antigen retrieval. The sections were permeabilized and blocked for 2 h with 2% normal donkey serum in PBS 0.2% Triton X‐100, and they were incubated at 4°C overnight with MT1 (Abcam), MT3 (Sigma), GFAP (Invitrogen), S100β (Abcam), MAP2 (Sigma), and TUJ1 (Biolegend) primary antibodies. Nuclear DNA was stained with 10 mg/mL DAPI (40043, Biotium) for 20 min. Finally, sections were submerged in 70% ethanol for 5 min and incubated with autofluorescence eliminator reagent for background removal. The preparation was mounted with ProlongTM Gold antifade mounting media (Invitrogen), and IF was evaluated with SP5 laser scanning confocal microscope (TCS SP5, Leica). Processing and analysis were performed on the maximal intensity projection of the z‐stack using Fiji software. Quantification was measured based on each protein positive signal percentage with respect to total nuclei in the DAPI channel.

### 2.7 RNA extraction and analysis by quantitative real‐time PCR

Total RNA extraction was performed by Trizol (Life Technologies). Reverse transcription was performed using random priming and Maxima First Strand cDNA Synthesis Kit (Thermo Fisher). To analyze gene expression, quantitative real‐time polymerase chain reaction (qRT‐PCR) with 20 ng of cDNA was performed by Absolute SYBR Green mix (Thermo Scientific) on a CFX384 thermal cycler (Bio‐Rad). Transcript levels were normalized to Glyceraldehyde‐3‐phosphate dehydrogenase (GAPDH) and measured using the ^ΔΔ^Ct relative quantification method.

### 2.8 Western blot analysis

Immunoblots were performed following standard procedures. Specific antibodies against MT1 (Abcam), MT3 (Sigma), and β‐actin (Sigma‐Aldrich) were used in the study. Detection was performed by chemiluminiscence using NOVEX ECL Chemi Substrate and SuperSignal™ West Femto Maximum Sensitivity Substrate (ThermoFisher).

### 2.9 Data analysis

Statistical analyses and graphics were performed using Microsoft Office Excel, IBM SPSS Statistics 20 and GraphPad Prism 8 software. Data are represented as mean values ± SEM, with the number of experiments (*n*) carried out for each assay. Unless otherwise indicated, statistical significance was calculated by Student's *t*‐test; ^≠^ *p* < 0.1, *p ≤ 0.05, **p ≤ 0.01, ***p ≤ 0.001. For correlation analysis, we first performed a Kolmogorov‐Smirnov test for the assessment of normality, and then we used Pearson's coefficient when samples were normally distributed or Spearman's coefficient when they were not normally distributed.

### 2.10 Ethics approval

Families authorized the use of human samples for research. This study was approved by the Clinical Research Ethics Committee of the Donostia University Hospital (protocol AMF‐EGM‐2016‐01) and adhered to the tenets of the Declaration of Helsinki.

## 3 RESULTS

### 3.1 Transcriptome analysis reveals differential gene expression patterns in centenarian hippocampus

**FIGURE 1:**
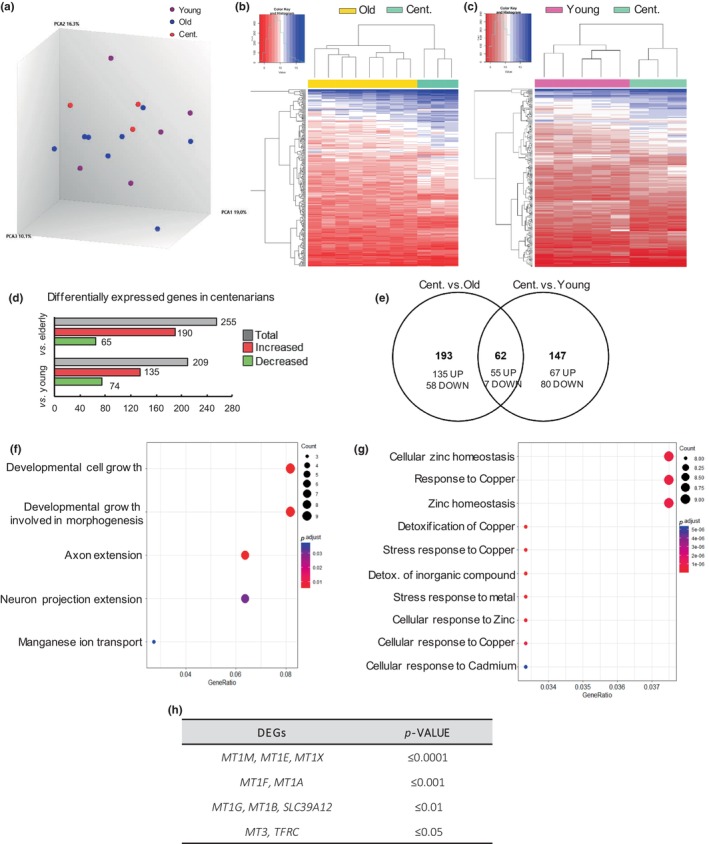
Differentially expressed genes in centenarians (≥97 years) hippocampus. (a). Principal component analysis (PCA) plot based on the expression profile of all transcripts measured. (b, c) Hierarchical clustering of centenarian (*n* = 3) versus old (*n* = 8) and young individuals (*n* = 5). (d) The number of genes increased and decreased genes in the transcriptomic analysis (*p*‐value < 0.05 and FC ≥ |2|). (e) Venn diagram showing the number of overlapping differentially expressed genes between the two comparisons. (f) Representative dotplot after GO analysis associated with decreased genes in centenarians versus old and young individuals. (g) Representative dot plot after Gene Ontology (GO) analysis associated with increased genes in centenarians versus old and young individuals. (h) Statistical significance of the genes associated with the biological pathways obtained after GO in (g).

PCA plot based on the expression profile of all analyzed genes in hippocampus from individuals of different ages showed that samples are not clustered (Figure 1a). On the other hand, the heatmap of DEGs and hierarchical clustering analysis of samples based on the expression profile of DEGs showed a separation between age groups and a differential gene expression pattern in centenarians compared to old (Figure 1b) and young individuals (Figure 1c). In particular, 255 genes were differentially expressed in centenarians compared to older individuals among which 190 were increased and 65 reduced (Tables S1 and S2). Moreover, 209 genes were differentially expressed in centenarians relative to young ones (Figure 1d) among which 135 were elevated and 74 decreased (Tables S3 and S4). Of note, 62 DEGs were shared in both analyses with majority of them increased **(**Figure 1e). GO analysis revealed that cell growth or axon extension processes were associated with downregulated genes in centenarians compared to the other two groups (Figure 1f and Figure S1A). On the other hand, heavy metal‐related biological processes were enriched in the genes upregulated in centenarians compared to the other two groups (Figure 1g). In detail, genes associated with pathways involved in zinc (Zn) homeostasis, detoxification of metals, and cellular response to metals were highly enriched (Figure 1g). The DEG associated with these biological processes included members of the metallothioneins (*MTs*) family, which were the ones most significantly increased in the hippocampus of centenarians (Figure 1h and Figure S1B). Moreover, the expression of *Transferrin Receptor* (*TFRC*) and *Solute Carrier Family 39 Member 12* (*SLC39A12*), also known as *ZIP12*, were also augmented (Figure 1e). *TFRC* is a cell surface receptor necessary for cellular iron uptake by the process of receptor‐mediated endocytosis that is required in neurologic development (Moos et al., 1998), and *ZIP12* is a zinc transporter with an important role in nervous system development, and its mutations or lower levels have been associated with several brain diseases (Chowanadisai et al., 2013). These results reveal a set of unique genes expressed in the hippocampus of centenarians.

### 3.2 MTs are highly expressed in the hippocampus of very old individuals

**FIGURE 2:**
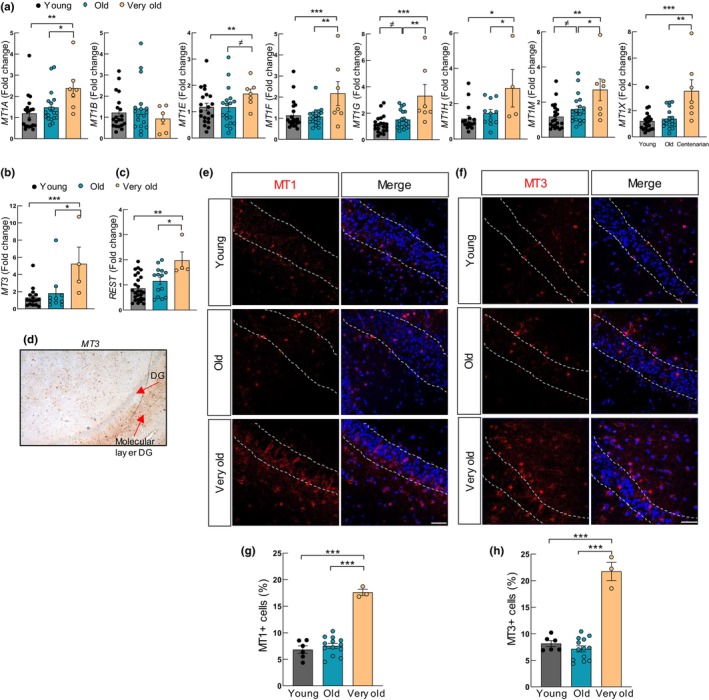
MTs are highly expressed in the hippocampus of very old individuals. (a, b) mRNA levels of indicated MT isoforms in hippocampus samples of young (*n* ≥ 16), elderly (*n* ≥ 10), and very old individuals (*n* ≥ 4) from cohort 1. (c) Expression of REST in same samples. (d) Representative immunohistochemistry image of MT3 in human hippocampus. (e, f) Representative immunofluorescence (IF) images of MT1 and MT3 (red) in the DG of young (*n* = 7), old (*n* = 14), and very old individuals (*n* = 3) from cohort 2 (scale bar = 50 μm). (g, h) Quantification of MT1 and MT3 protein levels in samples from cohort 2. The figure represents the percentage of MT1‐ or MT3‐positive cells with respect to nuclei stained with DAPI (blue). The statistical significance was assessed with the Student's *t*‐test (^≠^ *p* < 0.1, *p < 0.05, **p < 0.01, ***p < 0.001).

We further characterized *MTs* expression in hippocampus samples at mRNA levels and protein levels in two independent cohorts. In the first case, higher expression levels of mRNA of multiple *MT1* isoforms were detected in very old individuals (over 90 years old) compared to young and old individuals. Specifically, very old brain samples displayed a 2‐fold increase of *MT1A*, *MT1E*, *MT1F*, *MT1G*, *MT1H*, *MT1M*, and *MT1X* compared to young or old (Figure 2a). Similarly, the brain‐specific *MT3* isoform was significantly increased in very old individuals (Figure 2b). Additionally, the expression of *REST*, which has been described as an important player of extreme longevity and cognitive activity (Zullo et al., 2019), was elevated in hippocampus samples of very old individuals (Figure 2c). We also studied the mRNA expression of *MTs* divided by gender, but the low number of samples did not allow us to reach robust conclusions (Figure S2). In order to study the possibility of brain region specificity, the expression of different *MTs* subtypes was measured in cortex samples of individuals from the transcriptome analysis, and we did not detect significant changes in *MT* levels within very old and old individuals (Figure S3A). Consistent with the relevance of MTs in hippocampus, Allen Mouse Brain Atlas (https://mouse.brain‐map.org/) shows enrichment of MT1 and MT3 expression in hippocampus (particularly in the DG) (Figure S3B), and immunohistochemistry (IHC) revealed MT staining in the different regions of the human hippocampus (Figure 2d). IF analysis of DG in very old individuals also revealed significantly higher protein expression of MT1 and MT3 compared to the other two groups (Figure 2e–h). An additional analysis was performed to identify a possible correlation between MTs expression and age in the hippocampus samples from the two cohorts analyzed at mRNA and protein levels. Herein, we found that the expression of different *MT1* isoforms and *MT3* (Figure S3C,D) correlated positively with age at the mRNA level. However, there was no statistically significant correlation between MT1/MT3 protein levels and the age of individuals (Figure S3E). These results confirm that MTs are highly expressed in the hippocampus of very old individuals.

### 3.3 MTs are mainly expressed by astrocytes

**FIGURE 3:**
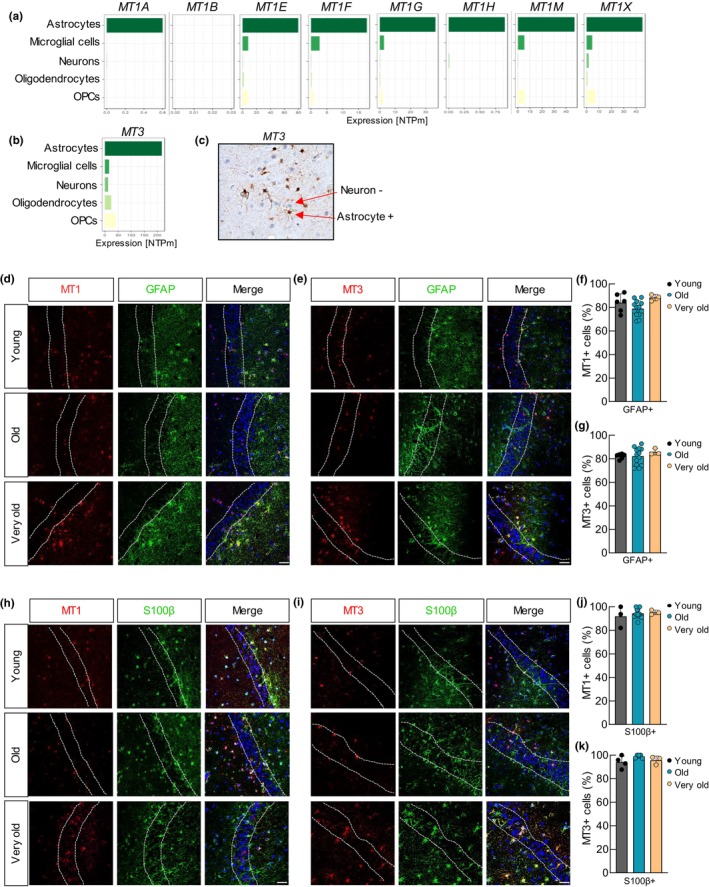
MTs are mainly expressed by astrocytes. (a, b) Normalized expression (nTPM) of MT1 and MT3 using single‐cell RNAseq data from the whole brain from “The Human Protein Atlas” (http://www.proteinatlas.org). (c) Immunohistochemistry of MT3 in human DG. (d) Representative immunofluorescence (IF) images of GFAP (green) and MT1 (red) in the dentate gyrus (DG, scale bar = 50 μm) of hippocampal coronal sections of young (*n* = 7), old (*n* = 14) and very old individuals (*n* = 3) from the cohort 2. (e) Representative IF images of GFAP (green) MT3 (red) in DG samples of the same cohort (young *n* = 7, old *n* = 14, very old *n* = 3; scale bar = 50 μm). (f, g) Quantification (percentage) of MT1‐ and MT3‐positive cells that are also positive for GFAP (MT1+/GFAP+ or MT3+/GFAP+ cells) in the DG of the same samples. (h) Representative IF images of S100β (green) and MT1 (red) in DG samples from cohort 2 (young, *n* = 3; elderly, *n* = 11; very old, *n* = 3; scale bar = 50 μm). (i) Representative IF images of S100β (far red) and MT3 (red) in DG samples of the same cohort (young, *n* = 4; old, *n* = 5; very old, *n* = 3; scale bar = 50 μm). (j, k) Quantification (percentage) of MT1‐ or MT3‐positive cells that are also positive for S100β (MT1+/S100β+ or MT3+/S100β+ cells) in the DG of the same samples.

In order to take a further step in the characterization of the expression of MTs, we studied their expression in different brain cell types. For this, we first analyzed single‐cell RNAseq studies performed in human brains from The Human Protein Atlas (http://www.proteinatlas.org). In this case, we observed that the different isoforms of *MT1* and *MT3* were expressed almost exclusively by astrocytes (Figure 3a, b). An additional RNAseq study of human astrocytes compared to other cell types of the brain (Zhang et al., 2016) showed similar results (Figure S4A,B). In line with this, IHC in human hippocampus samples indicated positive staining of MT3 in astrocytes (Figure 3c). To further study the link of *MTs* with astrocytes, co‐IF of both MT1 and MT3 with glial fibrillary acidic protein (GFAP) and S100 calcium‐binding protein β (S100β) were completed. These analyses revealed that on average, 80%–85% of the cells positive for MT1 or MT3 were also positive for GFAP (Figure 3d–g and Figure S4C). In the same line, co‐staining of MT1 or MT3 with S100β showed that 95% of MT‐positive cells were also positive for the astrocytic marker (Figure 3h–k and Figure S4D). These results were obtained in the different age groups, such as young, old, and very old. We also noted, as previously described (Salas et al., 2020; Zullo et al., 2019), that the number of GFAP‐positive cells increased with age (Figure S4E), and REST expression was neuronal as did not colocalize with GFAP but with Class III β‐Tubulin (TUJ1) (Figure S5A,B).

Next, we further characterized MTs expression in different cell types and performed co‐staining studies of MT1 and MT3 with markers of neurons. Co‐IF of both MT1 and MT3 with microtubule‐associated protein 2 (MAP2) and TUJ1 neuronal markers revealed that around 5% of the cells that were positive for MT1 or MT3 were also positive for MAP2 (Figure S6A–D) or TUJ1 (Figure S6E–H), independently of the age of the cases. We also checked their expression in microglia taking advantage of RNAseq data from aged human bulk dorsolateral prefrontal cortex samples and purified microglia from the same region (http://shiny.maths.usyd.edu.au/Ellis/MicrogliaPlots/). This analysis showed that none of the *MT1s* or *MT3* was enriched in microglia compared to the bulk cortex (Figure S6I,J). Together, these results reveal that astrocytes express high levels of MTs.

### 3.4 MTs expression decreases in cultured astrocytes

**FIGURE 4:**
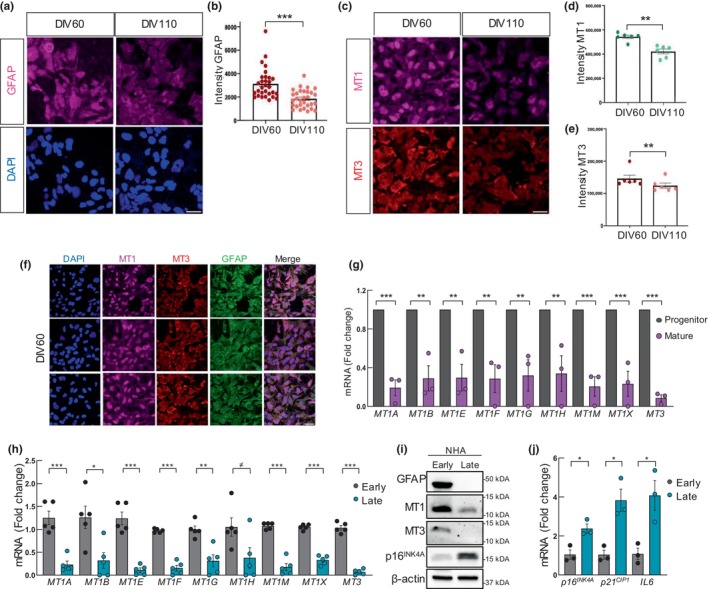
MTs expression decreases in cultured human astrocytes. (a, b) Representative immunofluorescence (IF) images and quantification of GFAP expression in hPSC‐derived astrocyte progenitors (day‐in vitro 60) and more mature astrocytes (day‐in vitro 110). Scale bar = 50 μm. (c) Representative IF images of MT1 and MT3 expression in astrocytes at DIV60 versus DIV110 (*n* = 3). Scale bar = 50 μm. (d, e) Quantification of the mean intensity of MT1 and MT3 in astrocytes at DIV60 and DIV110 (*n* = 3). (f) Representative images of GFAP, MT1, and MT3 in hPSC‐derived astrocytes at DIV60 (*n* = 3). Scale bar = 50 μm. (g) mRNA levels of *MT* isoforms in astrocytes at DIV53‐62 (progenitor) and DIV104‐110 (mature). (h) mRNA levels of *MT* isoforms in normal human astrocytes (NHA) cultured at early (1–3) and late (12–18) passages (*n* = 5). (i) Representative Western Blot of GFAP, MT1, MT3, and *p16* ^*INK4A*^ in NHA at early and late passage (*n* = 3). (j) Expression of *p16* ^*INK4A*^, *p21* ^*CIP1*^, *IL6* in NHA cultured at early (2, 3) and late (15–17) passages (*n* = 3). The statistical significance was assessed with the Student's *t*‐test (^≠^ *p* < 0.1, *p < 0.05, **p < 0.01, ***p < 0.001).

Since MTs are mainly expressed in astrocytes, we characterized the expression of MTs specifically in human astrocytes performing in vitro experiments. First, we measured their expression in astrocytes derived from human pluripotent stem cells (hPSCs) at two stages: as progenitors (DIV53‐62) and as mature astrocytes (DIV104‐110). This comparison showed a reduction in GFAP staining by IF and western blot analysis (Figure 4a,b and Figure S7A), which correlated with lower MT1 and MT3 protein levels in mature astrocytes at ≅ DIV110 compared to ≅DIV60 progenitors (Figure 4c–e and Figure S7A). Co‐IF confirmed the co‐staining of MT1 and MT3 with GFAP further linking MTs expression to astrocytes (Figure 4f). RT‐qPCR analysis further revealed a significant decrease of over 2.5‐fold in mRNA expression of all *MT1* isoforms and *MT3* in mature astrocytes after 110 days in culture (Figure 4g). We also compared the expression of *MTs* in NHA (human primary astrocytes) at early and late passages. In the case of MT1 protein and *MT1* mRNA subtypes, the expression was decreased in the majority of cases in late passage NHA, being statistically significant for *MT1A*, *MT1E*, *MT1F*, and *MT1X* (Figure 4h,i and Figure S7B). Similarly, the levels of MT3 and GFAP were also lower in late passage NHA (Figure 4h,i and Figure S7B,C). On the contrary, the markers of senescence and aging p16^INK4A^, *p21* ^*CIP1*^, and *IL6* were increased (Figure 4i,j). These results indicate that astrocytes maintained in culture for longer periods have lower levels of MTs.

### 3.5 MT1 and MT3 silencing impairs astrocytic homeostasis

**FIGURE 5:**
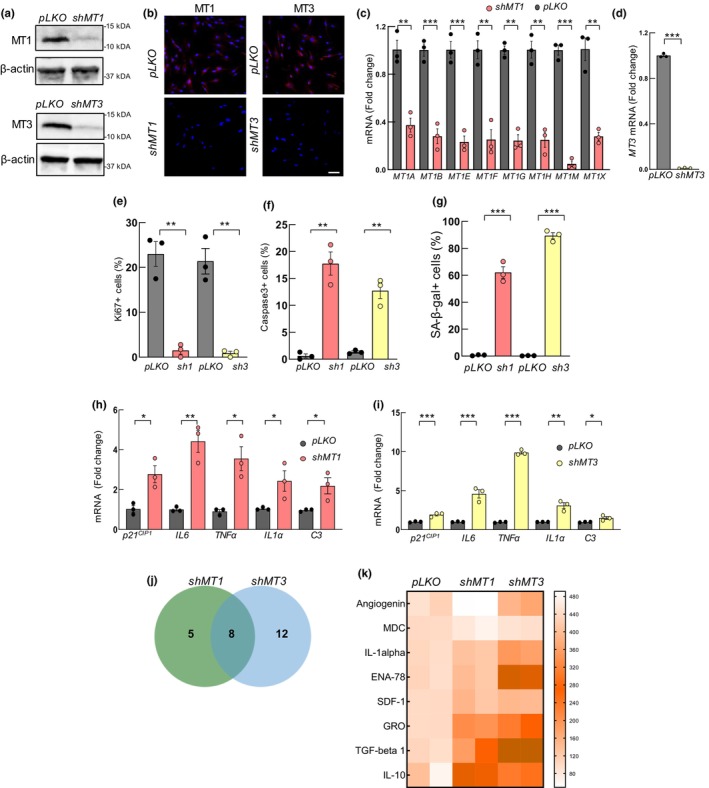
*MT1* and *MT3* silencing decrease astrocytic activity. *MT1* and *MT3* silencing was done in normal human astrocytes (NHA) using lentiviral infections. (a) Representative western blot of MT1 and MT3 in NHA with *MT1* (*shMT1*) or *MT3* silencing (*shMT3*) compared to controls (*pLKO*) (*n* = 3). (b) Representative immunofluorescence images of MT1 and MT3 in indicated cell types (scale bar = 50 μm). (c) mRNA levels of *MT1* isoforms in *shMT1* compared to *pLKO* cells (*n* = 3). (d) *MT3* levels in *shMT3* relative to *pLKO* (*n* = 3). (e) Quantification of Ki67^+^ cells, (f) Caspase 3^+^ cells, and (g) SA‐β‐gal activity in *MT* silenced compared to *pLKO* NHA (*n* = 3). (h, i) mRNA levels of *p21* ^*CIP1*^, *IL6*, *TNFα*, *IL1α*, and *C3* in *shMT1* and *shMT3* astrocytes compared to controls (*n* = 3). (j) Venn diagram and (k) heat map of the altered secreted common cytokines from supernatants of *shMT1* and *shMT3* NHA compared to *pLKO* astrocytes (*p* < 0.05, *n* = 2). The statistical significance was assessed with the Student's *t*‐test (^≠^ *p* < 0.1, *p < 0.05, **p < 0.01, ***p < 0.001).

We finally studied the possible effects of the modulation of *MTs* in NHA cells in order to unravel the possible role that MTs might have in astrocytes. For this, we independently silenced *MT1* and *MT3* in astrocytes using lentiviral infections and specific shRNA constructs for each gene. Western blot and IF revealed statistically significant lower protein levels and reduced number of cells expressing MT1 and MT3 in NHA with *MT1* or *MT3* downregulation (*shMT1* or *shMT3*) compared to control ones (*pLKO*) (Figure 5a,b and Figure S8A,B). In line with this, mRNA expression of all *MT1* subtypes and *MT3* was lower in *shMT1* and *shMT3* astrocytes, respectively (Figure 5c,d). Moreover, GFAP expression was also reduced in *shMT* cells (Figure S8B). Consequently, we performed different functional assays in order to analyze the cellular effects of *MT1* or *MT3* silencing. First, the cell growth study revealed that *MT*‐silenced NHA cells displayed different growth kinetics with lower cell numbers in *shMT1* or *shMT3* compared to control astrocytes measured by cell counting (Figure S8C). In line with this result, staining of Ki67 proliferation marker showed significantly reduced positive cells in *shMT1* and *shMT3* (from 20% in controls to less than 3% in *shMT* cells) (Figure 5e and Figure S8D). Furthermore, silencing of *MTs* significantly increased apoptosis measured as the number of positive cells for Caspase 3 marker (Figure 5f and Figure S8E). Finally, we also measured the effect of MTs silencing in cellular senescence, detecting a significantly higher number of SA‐β‐galactosidase positive cells in *shMT* cultures (Figure 5g and Figure S8F), as well as higher levels of *p21* ^*CIP1*^, cell cycle arrest and senescence marker, and *IL6*, SASP, and inflammation marker (Figure 5h,i). In summary, these results reveal that MT1 and MT3 have a role in astrocyte viability and activity.

Reactive astrocytes are activated in response to physiological and pathological states that cause morphological, molecular, and functional remodeling (Lawrence et al., 2023). Astrocyte reactivity is heterogenous, and two main subtypes have been proposed. Neuroprotective phenotype is related to promoting synaptic formation, growth of neurites, and production of anti‐inflammatory factors and cytotoxic or inflammatory that lead to cellular damage and are associated with aging, neurodegeneration, and disease. Based on this, we measured the expression of several pro‐inflammatory markers, such as TNFα, IL1α, and C3, linked to the cytotoxic subtype. All the markers were elevated at mRNA level in both *shMT1* and *shMT3* NHAs in comparison to control ones (Figure 5h,i). In order to further characterize the impact of *MTs* blockage, we studied the comprehensive profile of cytokines secreted by *shMT1* and *shMT3* NHA. For this, we performed a cytokine expression array from supernatants of control and *MTs* silenced cells. Interestingly, this study showed deregulated cytokines in both *MT*‐silencing cells with several cytokines and chemokines related to an inflammatory subtype of astrocytes (Figure S8G,H). Interestingly, eight were altered in both conditions (Figure 5j). Notably, secretion of IL1α, GRO (CXCL1), ENA78 (CXCL5), SDF1 (CXCL12), IL10, and TGFb1 was increased in *shMT* compared to control cells (Figure 5k). These results suggest that astrocytes with MT silencing acquire a cytotoxic profile.

## 4 DISCUSSION

We have performed a transcriptomic analysis in hippocampus samples of individuals of different ages, detecting a differential expression pattern in centenarians compared to young and elderly groups. Notably, heavy metal, in general, and Zinc‐related pathways, in particular, were GO terms enriched among differentially expressed transcripts in centenarians. There is a link between Zinc levels in the brain and cognitive function, and the decline in cognitive performance observed during aging or the apparition of neurodegenerative diseases, such as Alzheimer's disease (AD), have been linked to the dysregulation of Zinc homeostasis (Mocchegiani et al., 2006). For instance, reduced zinc transporter‐3 (ZnT3) expression or mutations of this gene, which is responsible for loading zinc into presynaptic vesicles in the hippocampus, have been associated with the onset of cognitive decline and accelerated brain aging, ultimately impairing learning and memory functions (Adlard et al., 2010; Saito et al., 2000). Additional metals, such as iron and copper, are also involved in brain aging since they trigger oxidative stress, accumulation of damaging molecules, DNA damage, and neuroinflammation (Ijomone et al., 2020).

The concentration of Zinc in the brain surpasses that of the body by 10‐fold and it is essential for normal functioning (Portbury & Adlard, 2017). It is tightly controlled, and it plays an important role in cellular and molecular processes, enzymatic activity, and regulation of transcription factors (Gower‐Winter & Levenson, 2012). In the brain, the majority of Zinc tightly binds to macromolecules such as proteins or amino acids and only 10%–15% exist as free Zinc (Gower‐Winter & Levenson, 2012). MTs, Zinc transporters family (ZnTs), presenilins, and zinc‐regulated and iron‐regulated proteins (ZIPs) are responsible for the homeostasis of Zinc in the brain (Portbury & Adlard, 2017). Among the DEGs, several members of the *MTs* family of genes were highly expressed in the hippocampus of very old individuals. *MTs* are a family of cysteine‐rich proteins involved in the homeostasis and detoxification of heavy metals, specifically, they bind divalent metals such as Zn and Cu (Babula et al., 2012). Different *MT1* subtypes have been described, which are found ubiquitously in a broad range of organs, in contrast to *MT3*, which is a brain‐specific isoform (Juárez‐Rebollar et al., 2017). They are not limited to the homeostasis or detoxification of heavy metals, and they have been described as having cytoprotective effects that promote cell survival and tissue regeneration. Indeed, they have been reported as anti‐inflammatory and antioxidants, with a role in the protection against oxidative stress, apoptosis, and DNA damage (Hidalgo et al., 2001; Juárez‐Rebollar et al., 2017). A European study focused on aging (MARK‐AGE project) showed that PBMCs from centenarian's offspring displayed increased basal expression of some *MTs* and *Zinc transporter 1* genes in comparison to the general population (Giacconi et al., 2018). In addition, *MTs* levels were found to be inversely related to oxidative stress markers (Giacconi et al., 2018). Moreover, an additional study performed in Italian centenarian females showed that a specific SNP corresponding to an Adenine/Cytosine (Asparagine/Threonine) transition at +647 nucleotide position in the *MT1A* coding region is associated with longevity since it was present in nonagenarian and centenarian females (Cipriano et al., 2006). In addition, it was associated with lower inflammatory status since lower levels of circulating IL6 in the plasma of these individuals were observed (Cipriano et al., 2006). Our results, together with these ones, indicate that centenarians and their offspring have enrichment of MTs and better control of Zn homeostasis that provides them protection against stress stimuli over the whole lifespan.

The results obtained in our study are further supported by studies in mouse models. Thus, a study performed in *Mt1/Mt2*‐null mice reported that *MT* mutants showed a poorer rate of learning and memory (Levin et al., 2006). Additionally, studies performed in *Mt1‐* or *Mt3*‐null mice models described that MTs have a role in CNS homeostasis since they are induced in the aging brain as a defensive mechanism to attenuate oxidative stress, ROS production, and inflammation (Carrasco et al., 2003; Thirumoorthy et al., 2011). Indeed, after damage caused by a focal cryolesion in the cortex, mice with deletions of *MTs* showed increased oxidative stress, apoptosis, astrogliosis, and secretion of several inflammatory cytokines, including TNFα, IL1α, and IL6 (Carrasco et al., 2003).

In addition, mice overexpressing *Mt1* were protected against mild focal cerebral ischemia, showing fewer infarcts and better functional recovery than the controls (van Lookeren et al., 1999), and the opposite was observed in null mice (Trendelenburg et al., 2002). Moreover, the protective role of high levels of MTs in additional organs with aging and longevity has also been documented. Thus, several studies have indicated that high levels of MTs prevent diabetic complications (Carlson et al., 2013), protect against organ damage during inflammation, and attenuate cardiac dysfunction (Sun et al., 2014). Regarding longevity, Mt1‐overexpressing mice model displayed increased lifespan (Malavolta et al., 2012). Consistently, cardiac‐specific MT transgenic mice also presented increased longevity together with an attenuation of oxidative stress (Yang et al., 2006). By contrast, *Mt1* knockout mice displayed shorter lifespan than wild‐type mice (Kadota et al., 2015).

Co‐staining studies of MT1 and MT3 with markers of specific cell types of the brain, single‐cell RNAseq from The Human Protein Atlas, and RNAseq study of progenitor and mature human astrocytes (Zhang et al., 2016) revealed that MTs were expressed mainly by astrocytes. Moreover, MT levels in astrocytes derived from human pluripotent stem cell lines were lower in mature astrocytes compared to progenitors. Physiological aging triggers astrocyte activation in order to respond to stress signals, and astrocyte dysfunction contributes to cognitive decline in aging (Salas et al., 2020). We found that old or late passage cultured human astrocytes presented lower levels of MTs and that silencing of *MT1* or *MT3* in human astrocytes, resulted in (i) decreased proliferation and (ii) increased apoptosis and senescence, indicating that MTs are involved in astrocytic homeostasis. These results are supported by studies performed in mouse models where the upregulation of *Mt3* in glial cells provided neuroprotective effect after brain damage acting as a growth inhibitory factor (Hidalgo, 2004). Moreover, studies performed in *Mt1* transgenic mice have shown that *Mt1* is expressed in response to brain damage and protects the CNS (Levin et al., 2006). In addition, we observed an increase in the expression of inflammatory genes and secreted cytokines after *MTs* silencing that could be linked with reactive phenotype of astrocytes (Clarke et al., 2018). Consistent with our data, additional studies have shown that MTs regulate the expression of inflammatory factors mainly cytokines such as IL6, TNFα, and interferons in the brain of mice (Penkowa et al., 2003; Poulsen et al., 2005). However, future studies would be necessary to identify whether MTs have a role in astrocyte polarization.

In summary, we detected high levels of MTs in the hippocampus of centenarian individuals. Moreover, MTs are expressed in astrocytes, where we confirmed that they have a role in their homeostasis maintenance. Our results suggest that MTs could respond to stressful situations and provide protection for the aging brain. Therefore, maintenance of their high levels could be a potential antiaging and cognitive decline strategy.

## AUTHOR CONTRIBUTIONS

A.S.‐A. performed the experiments, analyzed the results, and wrote the draft of the manuscript. P.R. and F.G. helped with the I.F. experiments and supervised experiments during their stay at the Francis Crick Institute. M.M.‐C., M.M.‐V., S.C.‐S., A.A., and D.O. completed the transcriptomic study, the consequent bionformatic analysis, and helped with the validations. L.R.‐B. and A.M.A. performed the experiments with iPSC‐derived astrocytes, and M.A. provided brain samples. All of them revised the manuscript. A.M. directed the project, obtained funds, contributed to data analysis, and wrote the draft of the manuscript.

## FUNDING INFORMATION

A.S‐A received predoctoral fellowships from the Instituto de Salud Carlos III (FI17/00250) and a Scientific Exchange Grant (STF9197) from the European Molecular Biology Organization (EMBO) to conduct the research in the Neural Stem Cell Biology laboratory (The Francis Crick Institute, London). S.C‐S received predoctoral fellowships from the Education Department of Basque Government and Spanish Association against Cancer (AECC—PRDGU246336CRUC). AA is supported by a postdoctoral fellowship from the Basque Government (POS_2020_1_0008). A.M lab is supported by grants from Instituto de Salud Carlos III and FEDER Funds (PI19/01355, DTS20/00179, and PI22/01905) Diputacion Foral Guipuzcoa—Adinberri (FADIN19/001, FA547/2022) and Health Department of the Basque Country (2022111069). A.M.A. is funded by the MCIN/AEI/10.13039/501100011033 (PID2021‐125443OB‐100 also by FEDER Una manera de hacer Europa and RYC2020‐029494‐I by FSE invierte en tu futuro), the Alzheimer's Association (AARG‐21‐850389), and the Basque Government (PIBA‐2020‐1‐0030).

## CONFLICT OF INTEREST STATEMENT

None.

## Supporting information


Figures S1–S8.

Tables S1–S4.

## Data Availability

The data discussed in this publication have been deposited in NCBI's Gene Expression Omnibus and are accessible in GSE201118.
