# Supplementary material for: Centenarian hippocampus displays high levels of astrocytic metallothioneins
Source: Aging Cell. 2024 May 20;23(8):e14201. doi: 10.1111/acel.14201 (PMC11320342; doi:10.1111/acel.14201)
Supplement: Supplementary file 1 — Figures S1–S8. [file ACEL-23-e14201-s002.pptx]

## Slide 1
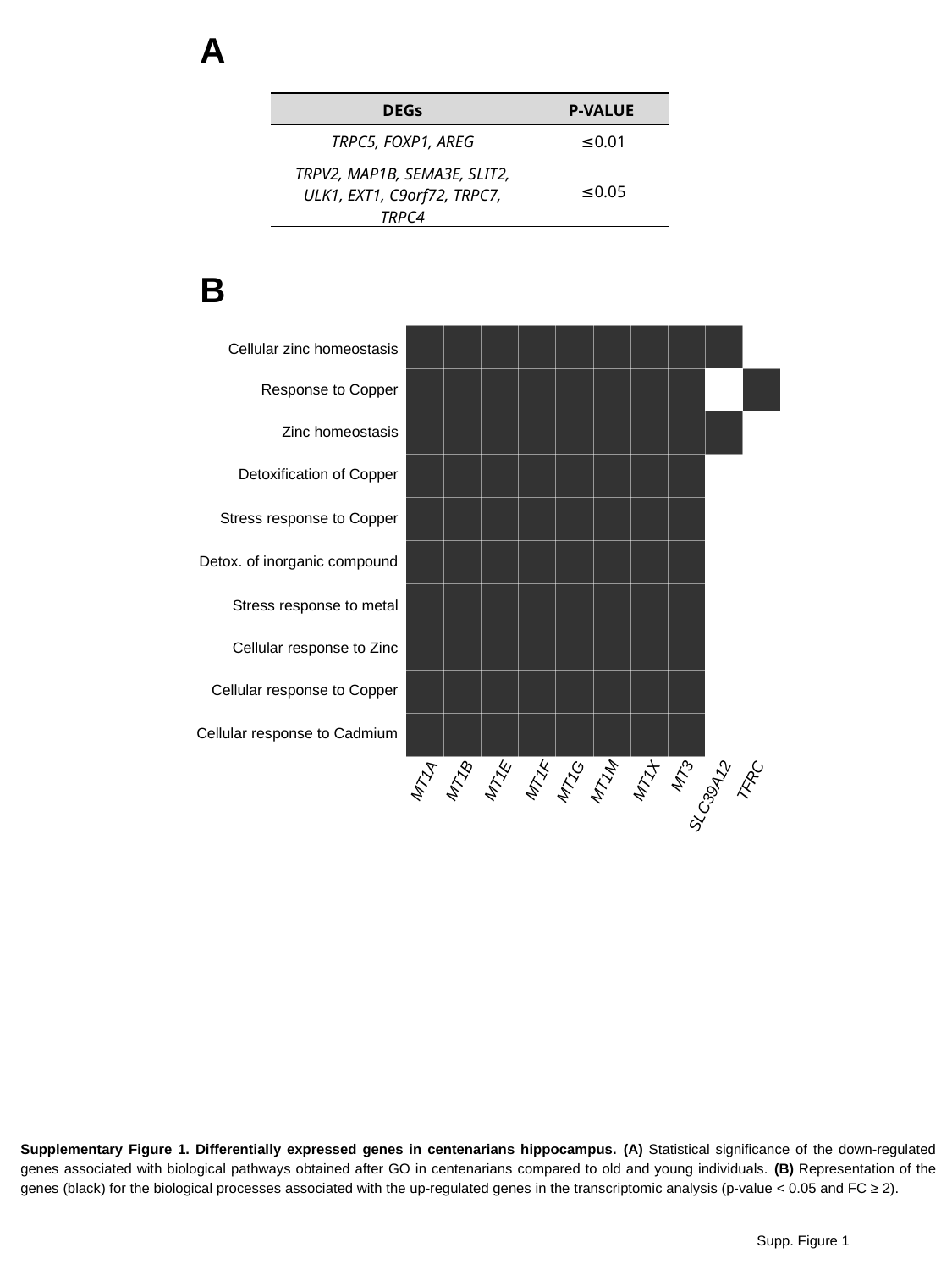

A
| DEGs | P-VALUE |
| --- | --- |
| TRPC5, FOXP1, AREG | ≤0.01 |
| TRPV2, MAP1B, SEMA3E, SLIT2, ULK1, EXT1, C9orf72, TRPC7, TRPC4 | ≤0.05 |
B
Cellular zinc homeostasis
Response to Copper
Zinc homeostasis
Detoxification of Copper
Stress response to Copper
Stress response to metal
Cellular response to Zinc
Cellular response to Copper
Cellular response to Cadmium
MT1G
MT1M
MT1X
MT3
MT1A
MT1B
MT1E
MT1F
SLC39A12
TFRC
Detox. of inorganic compound
Supplementary Figure 1. Differentially expressed genes in centenarians hippocampus. (A) Statistical significance of the down-regulated genes associated with biological pathways obtained after GO in centenarians compared to old and young individuals. (B) Representation of the genes (black) for the biological processes associated with the up-regulated genes in the transcriptomic analysis (p-value < 0.05 and FC ≥ 2).
Supp. Figure 1

## Slide 2
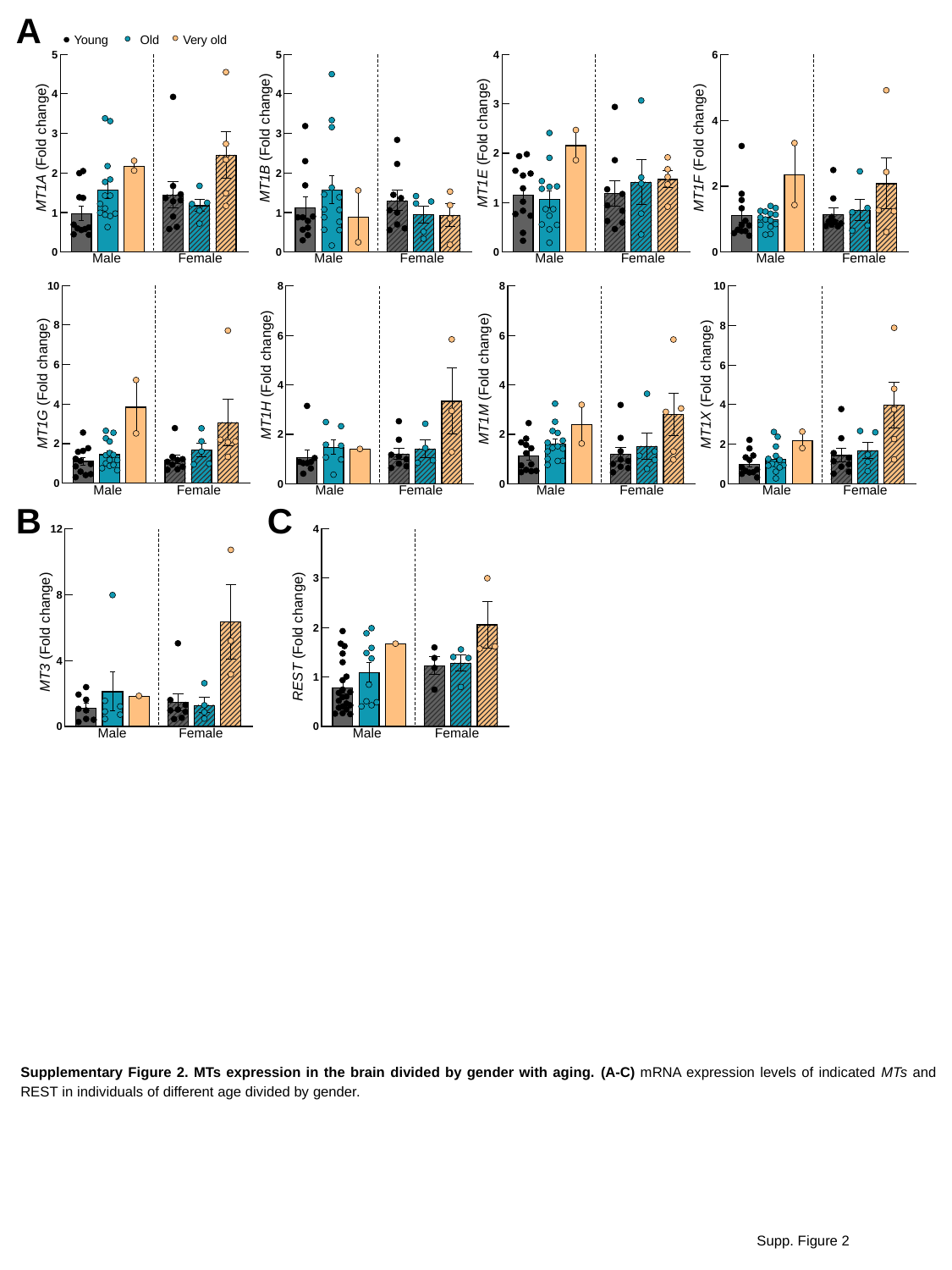

A
Young
Old
Very old
MT1B (Fold change)
MT1E (Fold change)
MT1A (Fold change)
MT1F (Fold change)
Male
Female
Male
Female
Male
Female
Male
Female
MT1H (Fold change)
MT1M (Fold change)
MT1G (Fold change)
MT1X (Fold change)
Male
Female
Male
Female
Male
Female
Male
Female
B
C
MT3 (Fold change)
REST (Fold change)
Male
Female
Male
Female
Supplementary Figure 2. MTs expression in the brain divided by gender with aging. (A-C) mRNA expression levels of indicated MTs and REST in individuals of different age divided by gender.
Supp. Figure 2

## Slide 3
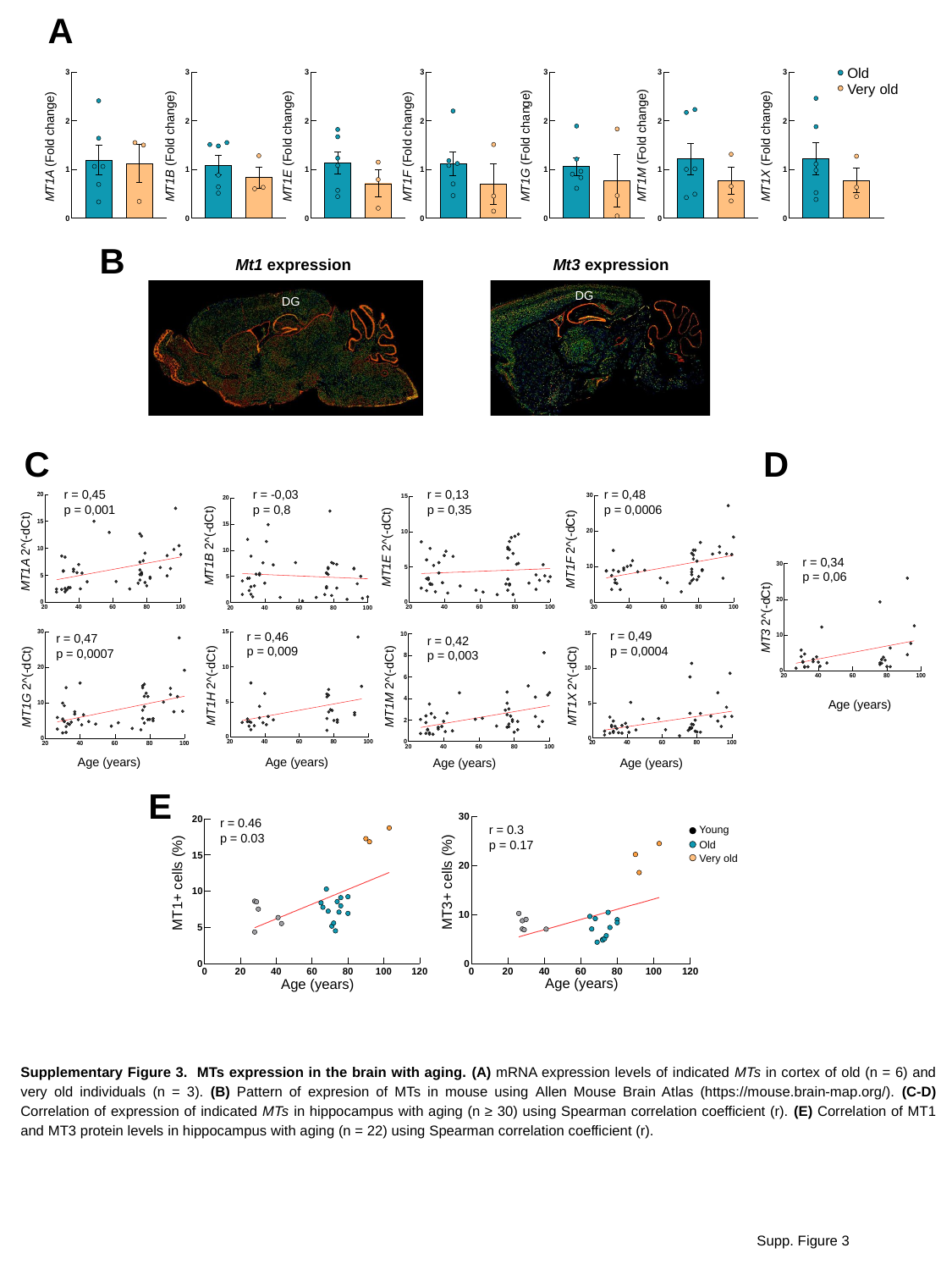

A
Old
Very old
MT1B (Fold change)
MT1F (Fold change)
MT1G (Fold change)
MT1A (Fold change)
MT1X (Fold change)
MT1E (Fold change)
MT1M (Fold change)
B
Mt1 expression
Mt3 expression
DG
DG
C
D
r = 0,45
p = 0,001
r = -0,03
p = 0,8
r = 0,13
p = 0,35
r = 0,48
p = 0,0006
MT1B 2^(-dCt)
MT1E 2^(-dCt)
MT1F 2^(-dCt)
MT1A 2^(-dCt)
r = 0,34
p = 0,06
MT3 2^(-dCt)
r = 0,49
p = 0,0004
r = 0,46
p = 0,009
r = 0,47
p = 0,0007
r = 0,42
p = 0,003
MT1X 2^(-dCt)
MT1M 2^(-dCt)
MT1H 2^(-dCt)
MT1G 2^(-dCt)
Age (years)
Age (years)
Age (years)
Age (years)
Age (years)
E
r = 0.46
p = 0.03
r = 0.3
p = 0.17
Young
Old
Very old
MT3+ cells (%)
Age (years)
MT1+ cells (%)
Age (years)
Supplementary Figure 3. MTs expression in the brain with aging. (A) mRNA expression levels of indicated MTs in cortex of old (n = 6) and very old individuals (n = 3). (B) Pattern of expresion of MTs in mouse using Allen Mouse Brain Atlas (https://mouse.brain-map.org/). (C-D) Correlation of expression of indicated MTs in hippocampus with aging (n ≥ 30) using Spearman correlation coefficient (r). (E) Correlation of MT1 and MT3 protein levels in hippocampus with aging (n = 22) using Spearman correlation coefficient (r).
Supp. Figure 3

## Slide 4
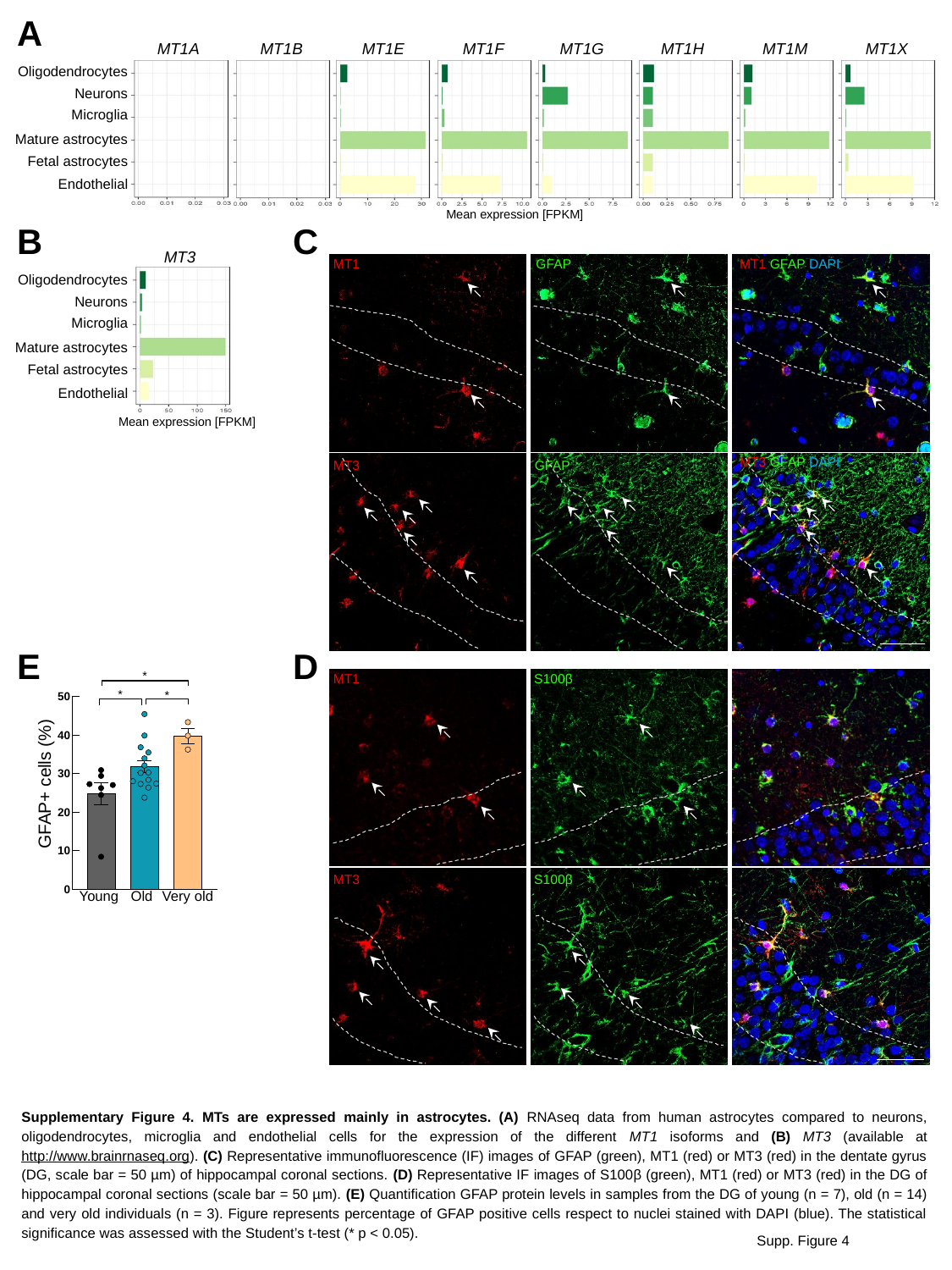

A
MT1A
MT1B
MT1E
MT1F
MT1G
MT1H
MT1M
MT1X
Oligodendrocytes
Neurons
Microglia
Mature astrocytes
Fetal astrocytes
Endothelial
Mean expression [FPKM]
B
C
MT3
Oligodendrocytes
Neurons
Microglia
Mature astrocytes
Fetal astrocytes
Endothelial
Mean expression [FPKM]
MT1
GFAP
MT1 GFAP DAPI
MT3 GFAP DAPI
MT3
GFAP
E
D
*
*
*
GFAP+ cells (%)
Young
Old
Very old
MT1
S100β
MT1 S100β DAPI
MT3
S100β
MT3 S100β DAPI
Supplementary Figure 4. MTs are expressed mainly in astrocytes. (A) RNAseq data from human astrocytes compared to neurons, oligodendrocytes, microglia and endothelial cells for the expression of the different MT1 isoforms and (B) MT3 (available at http://www.brainrnaseq.org). (C) Representative immunofluorescence (IF) images of GFAP (green), MT1 (red) or MT3 (red) in the dentate gyrus (DG, scale bar = 50 µm) of hippocampal coronal sections. (D) Representative IF images of S100β (green), MT1 (red) or MT3 (red) in the DG of hippocampal coronal sections (scale bar = 50 µm). (E) Quantification GFAP protein levels in samples from the DG of young (n = 7), old (n = 14) and very old individuals (n = 3). Figure represents percentage of GFAP positive cells respect to nuclei stained with DAPI (blue). The statistical significance was assessed with the Student’s t-test (* p < 0.05).
Supp. Figure 4

## Slide 5
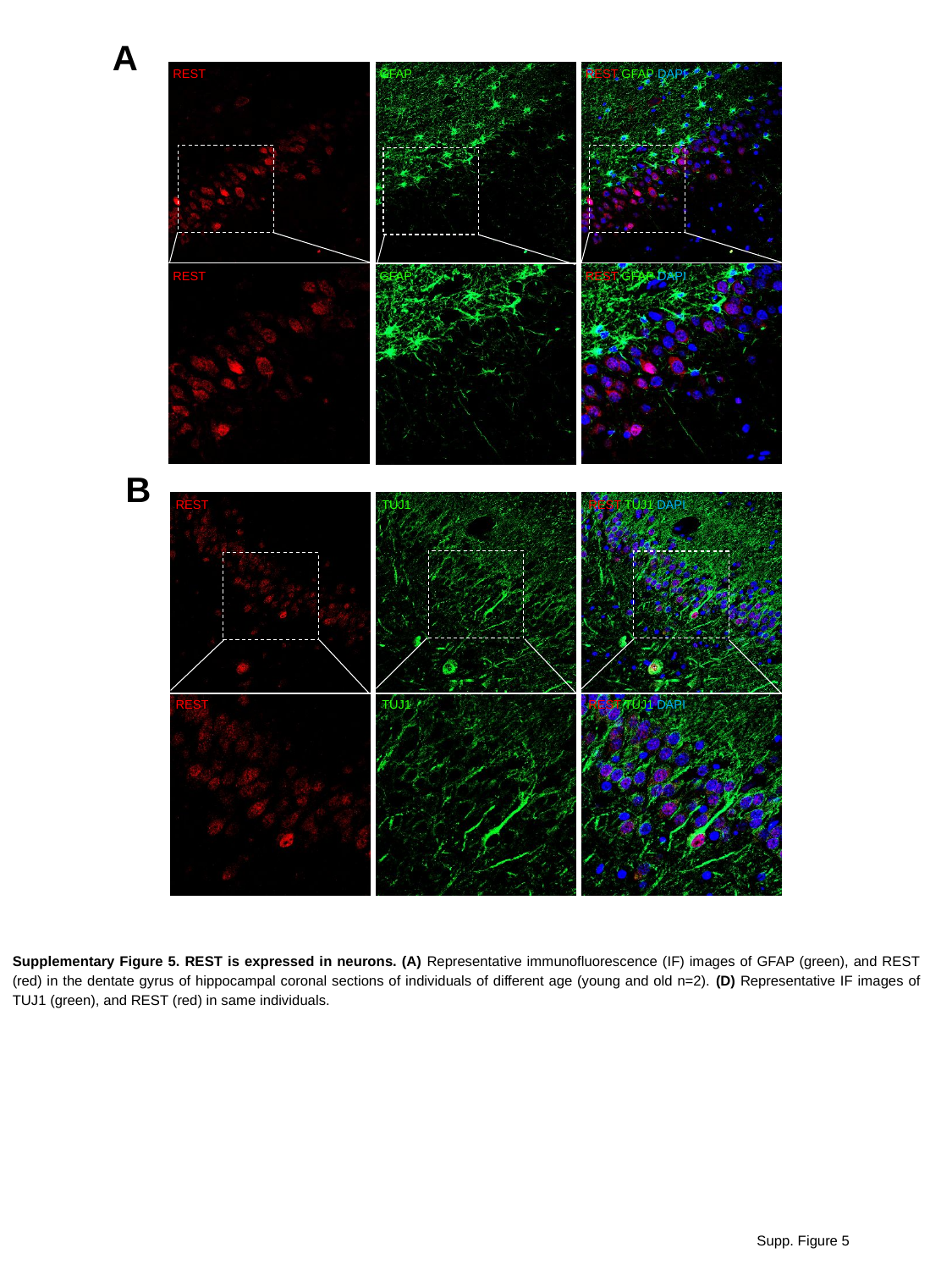

A
REST GFAP DAPI
GFAP
REST
REST GFAP DAPI
GFAP
REST
B
REST TUJ1 DAPI
TUJ1
REST
REST TUJ1 DAPI
TUJ1
REST
Supplementary Figure 5. REST is expressed in neurons. (A) Representative immunofluorescence (IF) images of GFAP (green), and REST (red) in the dentate gyrus of hippocampal coronal sections of individuals of different age (young and old n=2). (D) Representative IF images of TUJ1 (green), and REST (red) in same individuals.
Supp. Figure 5

## Slide 6
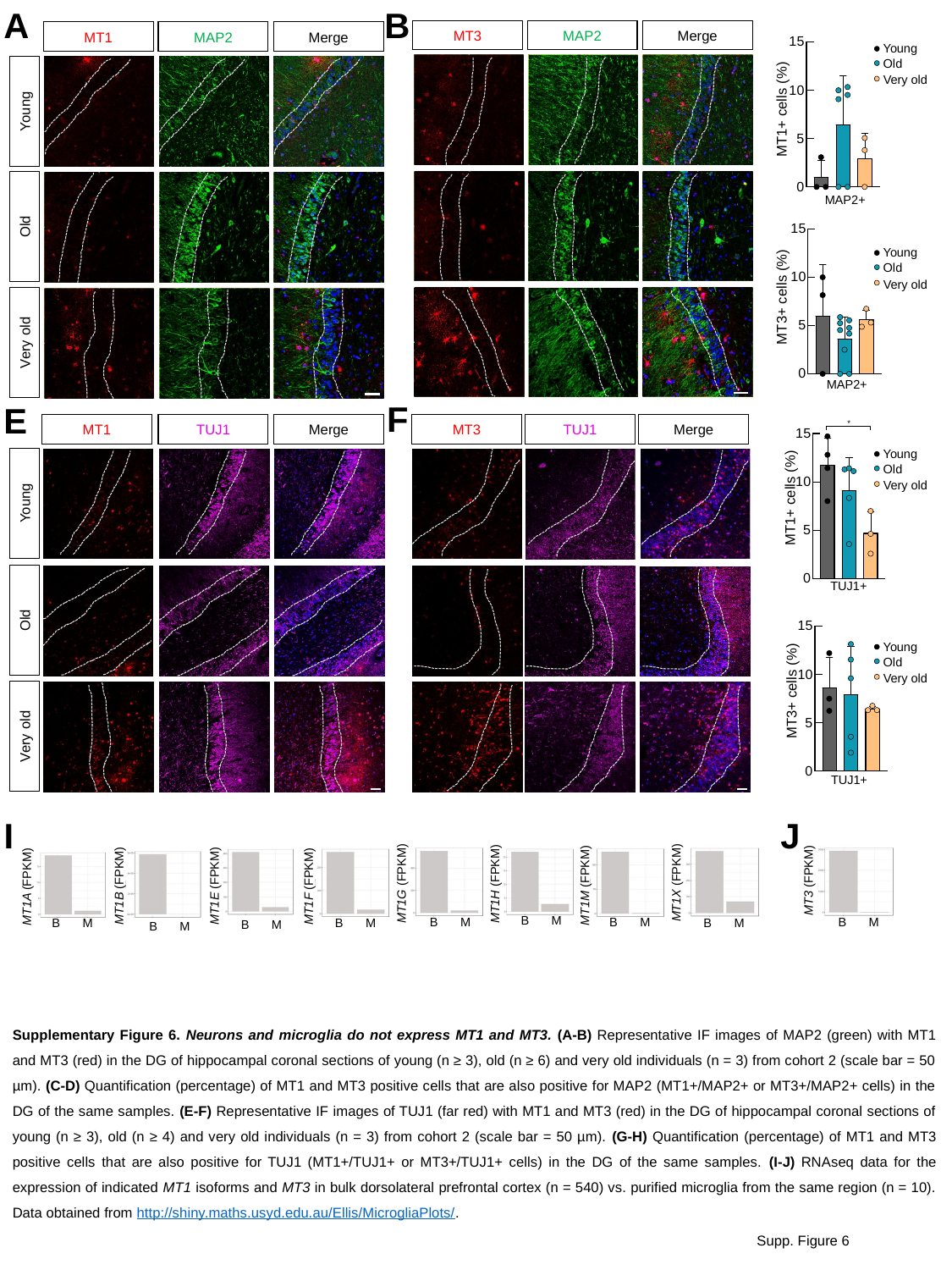

A
B
MT3
MAP2
Merge
MT1
MAP2
Merge
Young
Old
Very old
Young
Old
Very old
MT1+ cells (%)
MAP2+
MT3+ cells (%)
MAP2+
Young
Old
Very old
F
E
*
MT1
TUJ1
Merge
Young
Old
Very old
MT3
TUJ1
Merge
MT1+ cells (%)
TUJ1+
Young
Old
Very old
MT3+ cells (%)
TUJ1+
Young
Old
Very old
I
J
MT3 (FPKM)
B
M
MT1X (FPKM)
B
M
MT1G (FPKM)
B
M
MT1H (FPKM)
B
M
MT1B (FPKM)
B
M
MT1E (FPKM)
B
M
MT1F (FPKM)
B
M
MT1M (FPKM)
B
M
MT1A (FPKM)
B
M
Supplementary Figure 6. Neurons and microglia do not express MT1 and MT3. (A-B) Representative IF images of MAP2 (green) with MT1 and MT3 (red) in the DG of hippocampal coronal sections of young (n ≥ 3), old (n ≥ 6) and very old individuals (n = 3) from cohort 2 (scale bar = 50 µm). (C-D) Quantification (percentage) of MT1 and MT3 positive cells that are also positive for MAP2 (MT1+/MAP2+ or MT3+/MAP2+ cells) in the DG of the same samples. (E-F) Representative IF images of TUJ1 (far red) with MT1 and MT3 (red) in the DG of hippocampal coronal sections of young (n ≥ 3), old (n ≥ 4) and very old individuals (n = 3) from cohort 2 (scale bar = 50 µm). (G-H) Quantification (percentage) of MT1 and MT3 positive cells that are also positive for TUJ1 (MT1+/TUJ1+ or MT3+/TUJ1+ cells) in the DG of the same samples. (I-J) RNAseq data for the expression of indicated MT1 isoforms and MT3 in bulk dorsolateral prefrontal cortex (n = 540) vs. purified microglia from the same region (n = 10). Data obtained from http://shiny.maths.usyd.edu.au/Ellis/MicrogliaPlots/.
Supp. Figure 6

## Slide 7
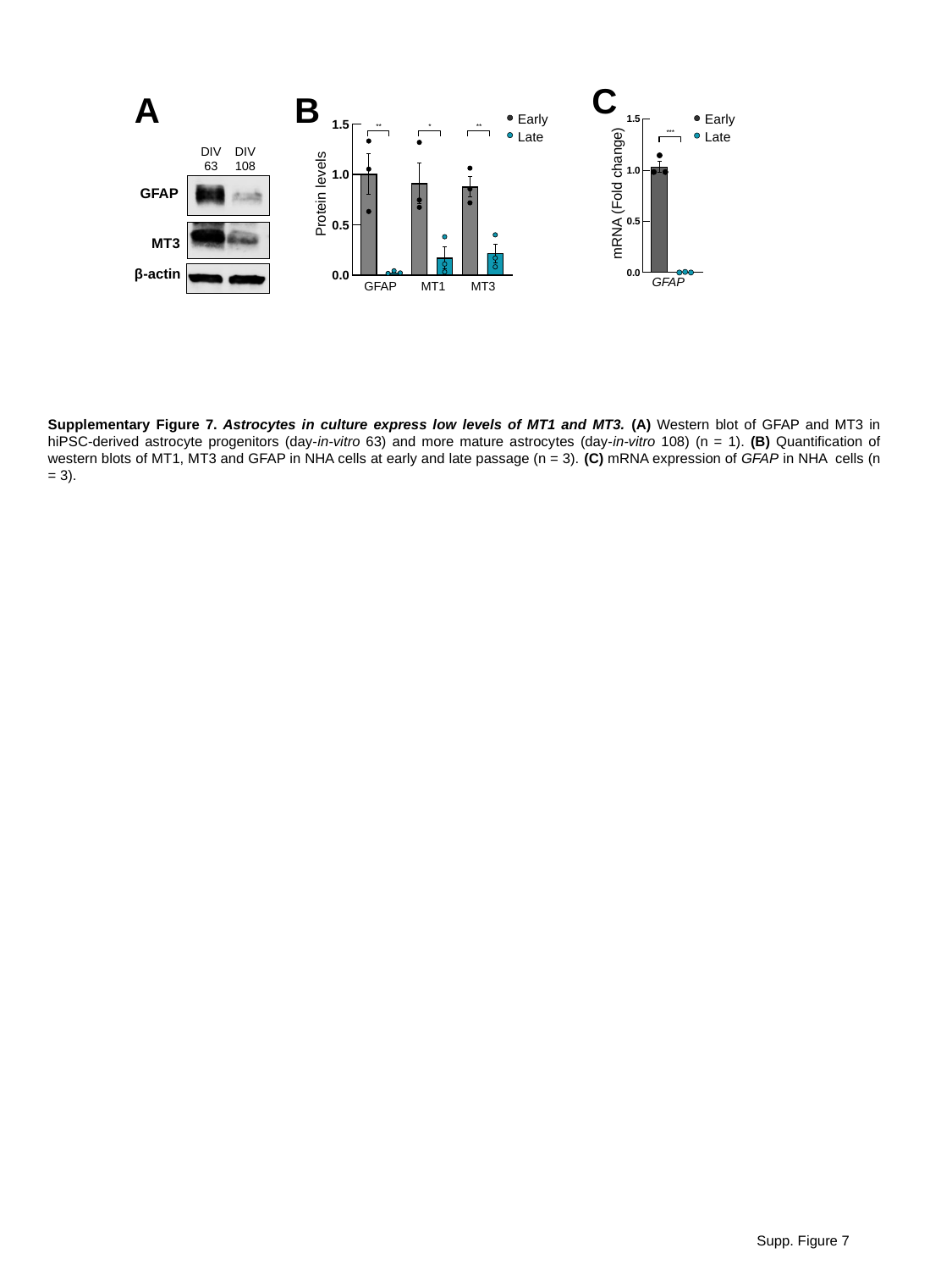

C
A
B
Protein levels
Early
Late
**
*
**
GFAP
MT1
MT3
Early
Late
***
DIV63
DIV108
mRNA (Fold change)
GFAP
MT3
β-actin
GFAP
Supplementary Figure 7. Astrocytes in culture express low levels of MT1 and MT3. (A) Western blot of GFAP and MT3 in hiPSC-derived astrocyte progenitors (day-in-vitro 63) and more mature astrocytes (day-in-vitro 108) (n = 1). (B) Quantification of western blots of MT1, MT3 and GFAP in NHA cells at early and late passage (n = 3). (C) mRNA expression of GFAP in NHA cells (n = 3).
Supp. Figure 7

## Slide 8
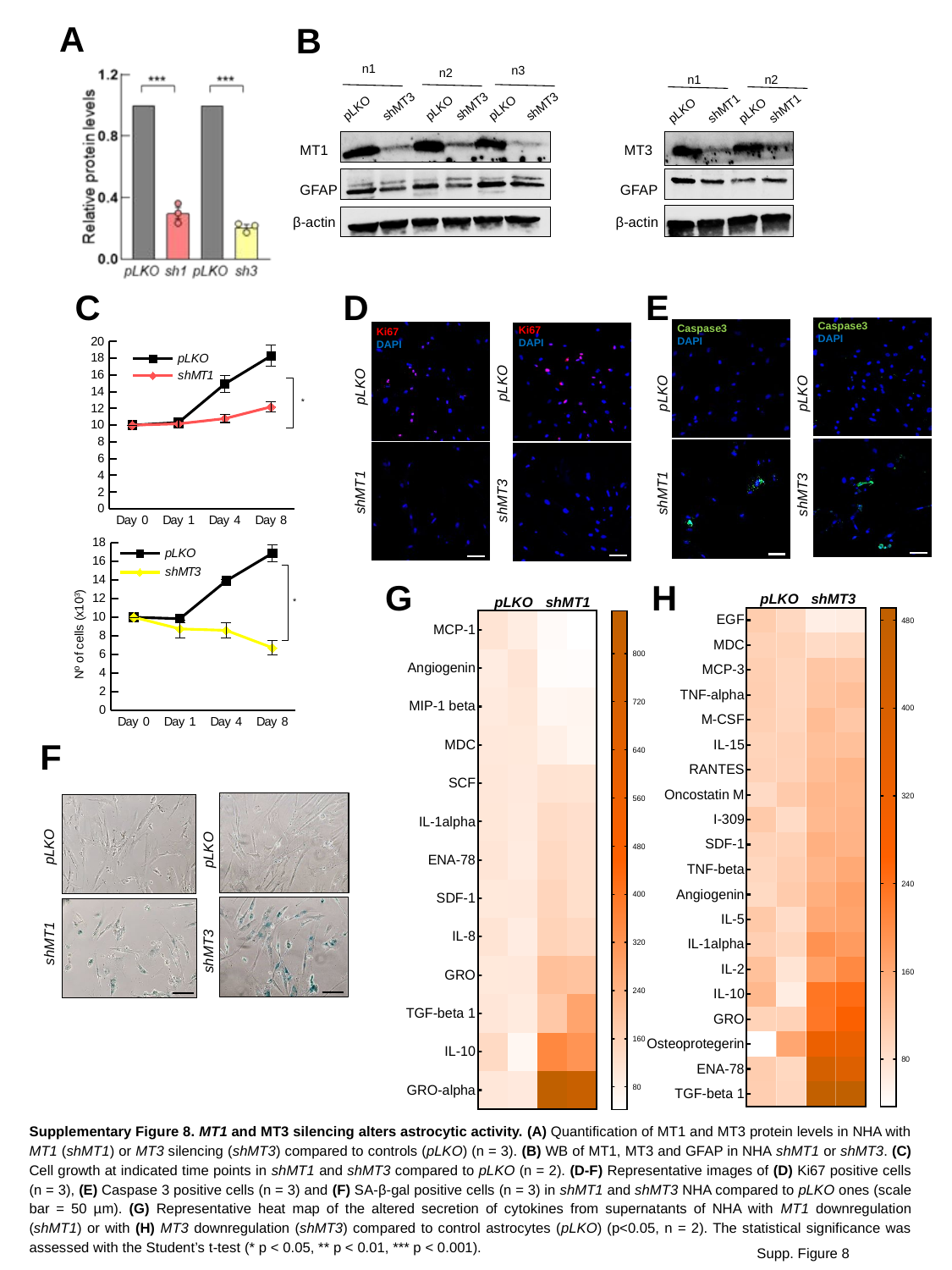

A
B
n1
n3
n2
n1
n2
shMT1
shMT1
pLKO
pLKO
shMT3
shMT3
shMT3
pLKO
pLKO
pLKO
MT1
MT3
GFAP
GFAP
β-actin
β-actin
D
E
C
Ki67
DAPI
pLKO
shMT3
Ki67
DAPI
pLKO
shMT1
Caspase3
DAPI
pLKO
shMT3
Caspase3
DAPI
pLKO
shMT1
### Chart
| Category | pLKO | shMT1 |
|---|---|---|
| Day 0 | 10.0 | 10.0 |
| Day 1 | 10.3125 | 10.15625 |
| Day 4 | 14.921875 | 10.78125 |
| Day 8 | 18.28125 | 12.1875 |
*
### Chart
| Category | pLKO | shMT3 |
|---|---|---|
| Day 0 | 10.0 | 10.0 |
| Day 1 | 9.84375 | 8.75 |
| Day 4 | 13.90625 | 8.59375 |
| Day 8 | 16.875 | 6.71875 |
G
H
pLKO
shMT3
pLKO
shMT1
*
Nº of cells (x103)
F
pLKO
shMT1
pLKO
shMT3
Supplementary Figure 8. MT1 and MT3 silencing alters astrocytic activity. (A) Quantification of MT1 and MT3 protein levels in NHA with MT1 (shMT1) or MT3 silencing (shMT3) compared to controls (pLKO) (n = 3). (B) WB of MT1, MT3 and GFAP in NHA shMT1 or shMT3. (C) Cell growth at indicated time points in shMT1 and shMT3 compared to pLKO (n = 2). (D-F) Representative images of (D) Ki67 positive cells (n = 3), (E) Caspase 3 positive cells (n = 3) and (F) SA-β-gal positive cells (n = 3) in shMT1 and shMT3 NHA compared to pLKO ones (scale bar = 50 µm). (G) Representative heat map of the altered secretion of cytokines from supernatants of NHA with MT1 downregulation (shMT1) or with (H) MT3 downregulation (shMT3) compared to control astrocytes (pLKO) (p<0.05, n = 2). The statistical significance was assessed with the Student’s t-test (* p < 0.05, ** p < 0.01, *** p < 0.001).
Supp. Figure 8
