# Supplementary material for: Centenarian hippocampus displays high levels of astrocytic metallothioneins
Source: Aging Cell. 2024 May 20;23(8):e14201. doi: 10.1111/acel.14201 (PMC11320342; doi:10.1111/acel.14201)
Supplement: Supplementary file 2 — Tables S1–S4. [file ACEL-23-e14201-s001.pdf]

Table 1. Genes increased in centenarians *vs* elderly in hippocampus

Gene symbol	Gene name	Fold Change	p-value
RASSF4	Ras association (RalGDS/AF-6) domain family member 4	2,57	0,000
MT1M	metallothionein 1M	3,41	0,000
ATP13A4	ATPase type 13A4	2,36	0,000
LRP4	LDL receptor related protein 4	2,44	0,000
LRRC8A	leucine rich repeat containing 8 family, member A	2,28	0,000
SLC25A18	solute carrier family 25, member 18	2,21	0,000
MT1E	metallothionein 1E	4,01	0,000
LRP6	LDL receptor related protein 6	2,02	0,000
CHD9	chromodomain helicase DNA binding protein 9	2,46	0,000
HEPACAM	hepatic and glial cell adhesion molecule	2,92	0,000
LGI4	leucine-rich repeat LGI family, member 4	2,72	0,000
MT1X	metallothionein 1X	4,22	0,000
ARHGAP31	Rho GTPase activating protein 31	2,74	0,000
MT1F	metallothionein 1F	3,1	0,001
FXYD1	FXYD domain containing ion transport regulator 1	5,83	0,001
PDGFRB	platelet-derived growth factor receptor, beta polypeptide	2,58	0,001
OPTN	optineurin	2,48	0,001
METTL7A	methyltransferase like 7A	4,71	0,001
MT1A	metallothionein 1A	2,58	0,001
TFCP2	transcription factor CP2	2,04	0,001
TPCN1	two pore segment channel 1	2,6	0,001
RAPGEF3	Rap guanine nucleotide exchange factor 3	2,27	0,001
ADCY2	adenylate cyclase 2	2,93	0,002
GPR75	G protein-coupled receptor 75	2,62	0,002
CTDSP1	CTD small phosphatase 1	3,08	0,002
GPR37L1	G protein-coupled receptor 37 like 1	2,52	0,002
SLC2A5	solute carrier family 2, member 5	2,65	0,002
SLC39A12	solute carrier family 39, member 12	2,65	0,002
ADGRG1	adhesion G protein-coupled receptor G1	3,61	0,002
CNTFR	ciliary neurotrophic factor receptor	3,18	0,002
HTRA1	HtrA serine peptidase 1	3,45	0,002
P3H2	prolyl 3-hydroxylase 2	4,13	0,002
ROCK1	Rho-associated, coiled-coil containing protein kinase 1	2,03	0,002
PHYHD1	phytanoyl-CoA dioxygenase domain containing 1	2,02	0,002
TNS2	tensin 2	2,01	0,002
MED12	mediator complex subunit 12	2,84	0,003
ALMS1	Alstrom syndrome protein 1	2,21	0,003
USP1	ubiquitin specific peptidase 1	2,17	0,003
DCHS2	dachsous cadherin-related 2	2,06	0,003
CABLES1	Cdk5 and Abl enzyme substrate 1	2,89	0,003
CLASP2	cytoplasmic linker associated protein 2	2,4	0,003
OPHN1	oligophrenin 1	2,05	0,004
SNTA1	syntrophin, alpha 1	2,11	0,004
ACACB	acetyl-CoA carboxylase beta	2,81	0,004
MERTK	MER proto-oncogene, tyrosine kinase	2,71	0,004
MT1G	metallothionein 1G	2,00	0,004

TP53BP2	tumor protein p53 binding protein 2	3,26	0,005
MAPRE1	microtubule-associated protein, RP/EB family, member 1	2,17	0,005
NPL	N-acetylneuraminate pyruvate lyase	3,22	0,005
TMEM176A	transmembrane protein 176A	2,14	0,005
TCF7L2	transcription factor 7-like 2 (T-cell specific, HMG-box)	2,52	0,006
IGFBP7	insulin like growth factor binding protein 7	3,46	0,006
PADI2	peptidyl arginine deiminase, type II	2,78	0,006
HOMEZ	homeobox and leucine zipper encoding	2,15	0,006
SLC14A1	solute carrier family 14, member 1	5,00	0,006
PAPLN	papilin, proteoglycan-like sulfated glycoprotein	3,49	0,006
PMS1	PMS1 homolog 1, mismatch repair system component	2,07	0,006
ADIRF	adipogenesis regulatory factor	2,71	0,006
PSD2	pleckstrin and Sec7 domain containing 2	2,02	0,006
GLUL	glutamate-ammonia ligase	2,16	0,007
MGLL	monoglyceride lipase	2,71	0,007
PRDX6	peroxiredoxin 6	2,32	0,007
TYRO3	TYRO3 protein tyrosine kinase	2,28	0,007
KCNN3	potassium channel, subfamily N alpha, member 3	3,27	0,007
STON2	stonin 2	2,51	0,007
PLXNB3	plexin B3	2,33	0,008
MT1L	metallothionein 1L	2,36	0,008
PNRC1	proline-rich nuclear receptor coactivator 1	2,38	0,008
CYP4V2	cytochrome P450, family 4, subfamily V, polypeptide 2	2,2	0,009
GJA1	gap junction protein alpha 1	3,34	0,009
EFHD1	EF-hand domain family member D1	2,11	0,009
MT1B	metallothionein 1B	2,22	0,011
CPM	carboxypeptidase M	2,13	0,011
SORL1	sortilin related receptor 1	2,35	0,011
FAT1	FAT atypical cadherin 1	2,46	0,011
STOM	stomatin	2,06	0,011
LIMCH1	LIM and calponin homology domains 1	2,03	0,011
SEC14L1	SEC14-like lipid binding 1	2,69	0,011
WASF2	WAS protein family, member 2	2,29	0,011
SEPN1	selenoprotein N, 1	2,13	0,012
CAPN2	calpain 2, (m/II) large subunit	2,14	0,012
ARHGEF4	Rho guanine nucleotide exchange factor 4	2,19	0,012
PFKFB3	6-phosphofructo-2-kinase/fructose-2,6-biphosphatase 3	3,06	0,012
RGCC	regulator of cell cycle	2,3	0,012
ZNRF3	zinc and ring finger 3	2,27	0,013
TMEM259	transmembrane protein 259	2,5	0,013
WIF1	WNT inhibitory factor 1	2,64	0,013
TPD52L1	tumor protein D52-like 1	2,64	0,013
FERMT2	fermitin family member 2	2,54	0,013
AMOT	angiomotin	2,48	0,013
PREX1	phosphatidylinositol-3,4,5-trisphosphate-dependent Rac exchange factor 1	2,29	0,013
HSDL2	hydroxysteroid dehydrogenase like 2	2,49	0,014
CHST11	carbohydrate sulfotransferase 11	2,22	0,014
HEPN1	hepatocellular carcinoma, down-regulated 1	2,74	0,014

PTCH1	patched 1	2,37	0,014
MDM4	MDM4, p53 regulator	2,14	0,015
KLF10	Kruppel-like factor 10	2,12	0,015
PAX6	paired box 6	3,02	0,015
LOC101929372	uncharacterized LOC101929372	2,69	0,015
CHPT1	choline phosphotransferase 1	2,85	0,016
AHCYL1	adenosylhomocysteinase like 1	2,44	0,016
PPP1R1B	protein phosphatase 1, regulatory subunit 1B	3,01	0,017
SHANK1	SH3 and multiple ankyrin repeat domains 1	2,17	0,017
TRPS1	trichorhinophalangeal syndrome I	2,22	0,017
ALG6	ALG6, alpha-1,3-glucosyltransferase	2,63	0,017
TAF15	TATA-box binding protein associated factor 15	2,36	0,018
GPRC5B	G protein-coupled receptor, class C, group 5, member B	2,78	0,018
GRAMD3	GRAM domain containing 3	2,47	0,018
ENG	endoglin	2,46	0,018
NEK11	NIMA-related kinase 11	2,00	0,019
CTSH	cathepsin H	2,94	0,019
GSTM5	glutathione S-transferase mu 5	2,83	0,021
DOCK7	dedicator of cytokinesis 7	2,1	0,021
PROX1	prospero homeobox 1	2,99	0,021
TBX3	T-box 3	2,84	0,021
PITPNC1	phosphatidylinositol transfer protein, cytoplasmic 1	2,3	0,021
GOLIM4	golgi integral membrane protein 4	2,58	0,021
SLC16A1	solute carrier family 16, member 1	2,51	0,021
XPNPEP3	X-prolyl aminopeptidase 3, mitochondrial	2,06	0,022
KCNJ10	potassium channel, inwardly rectifying subfamily J, member 10	2,69	0,022
GYS1	glycogen synthase 1	2,05	0,022
ATP1B2	ATPase, Na+/K+ transporting, beta 2 polypeptide	3,15	0,022
ETNPPL	ethanolamine-phosphate phospho-lyase	2,33	0,023
HBA1	hemoglobin, alpha 1	3,98	0,023
ARHGAP24	Rho GTPase activating protein 24	2,04	0,023
PDK4	pyruvate dehydrogenase kinase, isozyme 4	3,8	0,023
FAM198B	family with sequence similarity 198, member B	2,86	0,023
STAT3	signal transducer and activator of transcription 3	2,07	0,023
MRAS	muscle RAS oncogene homolog	2,02	0,024
EZR	ezrin	3,00	0,024
ZNF462	zinc finger protein 462	2,02	0,024
RFTN2	raftlin family member 2	2,27	0,025
PECAM1	platelet/endothelial cell adhesion molecule 1	2,22	0,025
GNA12	guanine nucleotide binding protein alpha 12	2,1	0,025
CLDN5	claudin 5	3,17	0,026
ING3	inhibitor of growth family member 3	2,29	0,026
TXNIP	thioredoxin interacting protein	2,46	0,026
HRSP12	heat-responsive protein 12	2,42	0,027
NPAS3	neuronal PAS domain protein 3	2,00	0,027
GIMAP7	GTPase, IMAP family member 7	2,06	0,028
TNKS	tankyrase, TRF1-interacting ankyrin-related ADP-ribose polymerase	2,18	0,028
PARP4	poly(ADP-ribose) polymerase family member 4	2,02	0,031

HBA2	hemoglobin, alpha 2	2,52	0,031
KCNJ16	potassium channel, inwardly rectifying subfamily J, member 16	2,03	0,032
NFKBIA	nuclear factor of kappa light polypeptide gene enhancer in B-cells inhibitor, alpha	3,08	0,033
FAXDC2	fatty acid hydroxylase domain containing 2	2,88	0,033
BMP2K	BMP2 inducible kinase	2,13	0,033
RANBP3L	RAN binding protein 3-like	4,23	0,034
BAMBI	BMP and activin membrane-bound inhibitor	2,33	0,034
S100A1	S100 calcium binding protein A1	2,26	0,034
EPS15	epidermal growth factor receptor pathway substrate 15	2,61	0,035
GRAMD1C	GRAM domain containing 1C	2,12	0,035
POLR2G	polymerase II polypeptide G	2,06	0,035
AAMDC	adipogenesis associated, Mth938 domain containing	2,45	0,036
MLXIP	MLX interacting protein	2,35	0,036
ARRDC3	arrestin domain containing 3	2,25	0,036
MTSS1L	metastasis suppressor 1-like	2,05	0,036
RPS6KA2	ribosomal protein S6 kinase, 90kDa, polypeptide 2	2,11	0,036
MECOM	MDS1 and EVI1 complex locus	2,01	0,037
EPAS1	endothelial PAS domain protein 1	3,33	0,037
CCNB1IP1	cyclin B1 interacting protein 1	2,7	0,038
ATP11C	ATPase, class VI, type 11C	2,45	0,038
NT5DC2	5-nucleotidase domain containing 2	2,44	0,038
EPHX1	epoxide hydrolase 1, microsomal	2,51	0,039
AGT	angiotensinogen	2,29	0,041
MT3	metallothionein 3	5,69	0,041
WFS1	Wolfram syndrome 1 (wolframin)	2,06	0,041
ITGB4	integrin beta 4	2,29	0,041
PCDHG	protocadherin gamma	2,28	0,041
FAM177B	family with sequence similarity 177, member B	2,43	0,041
NOTCH2NL	notch 2 N-terminal like	3,6	0,041
SDC4	syndecan 4	2,31	0,041
NUDT6	nudix hydrolase 6	2,64	0,042
PLPP3	phospholipid phosphatase 3	3,26	0,043
PODXL	podocalyxin-like	2,02	0,043
ZFP36L1	ZFP36 ring finger protein-like 1	3,5	0,044
CD58	CD58 molecule	2,71	0,044
SFMBT2	Scm-like with four mbt domains 2	2,24	0,045
CST3	cystatin C	2,15	0,045
PLEKHG1	pleckstrin homology domain containing, family G member 1	2,16	0,045
PYGL	glycogen phosphorylase L	2,22	0,045
SLC16A1	solute carrier family 16, member 1	2,06	0,046
RUBCN	RUN domain and cysteine-rich domain containing, Beclin 1-interacting protein	2,65	0,046
LHX2	LIM homeobox 2	2,42	0,047
RBBP8	retinoblastoma binding protein 8	2,05	0,047
SAFB2	scaffold attachment factor B2	2,08	0,047
BID	BH3 interacting domain death agonist	2,1	0,047
SLC2A1	solute carrier family 2, member 1	4,03	0,047
LHFP	lipoma HMGIC fusion partner	2,25	0,048
GMNN	geminin DNA replication inhibitor	2,05	0,049

Table 2. Genes decreased in centenarians *vs* eldery in hippocampus

Gene symbol	Gene name	Fold Change	p-value
POGZ	pogo transposable element derived with ZNF domain	-2,58	0,001
CCBE1	collagen and calcium binding EGF domains 1	-2,31	0,001
TDO2	tryptophan 2,3-dioxygenase	-2,06	0,001
PSMD13	proteasome 26S subunit, non-ATPase 13	-2,8	0,002
SPINK1	serine peptidase inhibitor, Kazal type 1	-2,04	0,002
PDS5B	PDS5 cohesin associated factor B	-2,11	0,003
SLC38A6	solute carrier family 38, member 6	-3,39	0,003
TRMT6	tRNA methyltransferase 6	-2,16	0,003
CD274	CD274 molecule	-2,79	0,004
TRPC5	transient receptor potential cation channel, subfamily C, member 5	-2,27	0,005
AREG	amphiregulin	-2,43	0,005
CHN2	chimerin 2	-2,02	0,005
ABCD2	ATP binding cassette subfamily D, member 2	-2,52	0,005
ARPP21	cAMP regulated phosphoprotein 21	-2,22	0,006
C3orf49	chromosome 3 open reading frame 49	-2,47	0,006
PANK1	pantothenate kinase 1	-2,04	0,007
SHCBP1	SHC SH2-domain binding protein 1	-2,42	0,008
PRG1	p53-responsive gene 1	-2,08	0,009
CLLU1OS	chronic lymphocytic leukemia up-regulated 1 opposite strand	-2,05	0,009
HCP5	HLA complex P5	-2,03	0,011
UTS2	urotensin 2	-2,01	0,011
TRPV2	transient receptor potential cation channel, subfamily V, member 2	-2,08	0,011
ADAT2	adenosine deaminase, tRNA-specific 2	-2,08	0,011
DEFB106A	defensin, beta 106A	-2,1	0,011
GBP3	guanylate Binding Protein 3	-2,29	0,011
PLCZ1	phospholipase C, zeta 1	-2,12	0,011
MAP1B	microtubule associated protein 1B	-2,14	0,012
OR4D1	olfactory receptor, family 4, subfamily D, member 1	-2,13	0,013
ZNF442	zinc finger protein 442	-2,09	0,013
PKP4	plakophilin 4	-2,02	0,017
PTPN20	protein tyrosine phosphatase, non-receptor type 20	-2,15	0,017
CLUL1	clusterin-like 1	-2,64	0,017
F11	coagulation factor XI	-2,24	0,017
PCDH11X	protocadherin 11 X-linked	-2,11	0,017
RPIA	ribose 5-phosphate isomerase A	-2,00	0,018
SLC12A5	solute carrier family 12, member 5	-2,12	0,018
KLHL11	kelch-like family member 11	-2,06	0,018
TBPL1	TBP-like 1	-2,69	0,019
CHI3L2	chitinase 3-like 2	-3,35	0,020
FAM19A1	family with sequence similarity 19 (chemokine (C-C motif)-like), member A1	-2,17	0,021
POLR2M	RNA polymerase II subunit M	-2,12	0,021
OR4C11	olfactory receptor, family 4, subfamily C, member 11	-2,35	0,022
PROS1	protein S	-2,24	0,023
OR51G1	olfactory receptor, family 51, subfamily G, member 1	-2,36	0,024
DEFB126	defensin, beta 126	-2,01	0,024

TRPC7	transient receptor potential cation channel, subfamily C, member 7	-2,04	0,024
HELB	helicase (DNA) B	-2,04	0,027
PFDN4	prefoldin subunit 4	-2,38	0,029
RNF17	ring finger protein 17	-2,45	0,031
EDDM3A	epididymal protein 3A	-2,22	0,031
TNNC1	troponin C type 1	-2,14	0,031
PRKG2	protein kinase, cGMP-dependent, type II	-2,03	0,031
TSPAN13	tetraspanin 13	-2,76	0,035
FAM151B	family with sequence similarity 151 member B	-2,83	0,036
LRRC28	leucine rich repeat containing 28	-2,5	0,037
LOC643802	u3 small nucleolar ribonucleoprotein protein MPP10-like	-2,01	0,038
FRG1BP	FSHD region gene 1 family member B	-2,62	0,041
CLC	Charcot-Leyden crystal galectin	-2,02	0,042
TRPC4	transient receptor potential cation channel, subfamily C, member 4	-2,1	0,043
LAMA1	laminin, alpha 1	-2,07	0,043
GPRASP2	G protein-coupled receptor associated sorting protein 2	-2,05	0,043
ZNF705G	zinc finger protein 705G	-2,45	0,043
SEMA3E	sema domain, immunoglobulin domain, secreted 3E	-2,34	0,044
SULT4A1	sulfotransferase family 4A member 1	-2,1	0,045
PATE2	prostate and testis expressed 2	-2,16	0,046

Table 3. Genes increased in centenarians *vs* young individuals in hippocampus

Gene symbol	Gene name	Fold Change	p-value
FASTKD3	FAST kinase domains 3	2,449	5,33E-05
ANKRD18B	ankyrin repeat domain 18B	4,537	0,000
RASGEF1B	RasGEF domain family member 1B	2,770	0,000
CCDC84	coiled-coil domain containing 84	4,071	0,000
LOC101929372	uncharacterized LOC101929372	3,027	0,001
FAM177B	family with sequence similarity 177, member B	3,687	0,001
CXorf57	chromosome X open reading frame 57	2,254	0,002
RASSF4	Ras association domain family member 4	2,174	0,002
PLXNB3	plexin B3	3,689	0,002
GPR37L1	G protein-coupled receptor 37 like 1	2,838	0,003
PADI2	peptidyl arginine deiminase, type II	4,019	0,003
LRRC1	leucine rich repeat containing 1	2,115	0,003
PREX1	phosphatidylinositol-3,4,5-trisphosphate-dependent Rac exchange factor 1	3,611	0,004
SLC48A1	solute carrier family 48 (heme transporter), member 1	2,001	0,004
CHI3L1	chitinase 3-like 1	3,116	0,004
CPB2	carboxypeptidase B2	2,256	0,004
ARAP2	ArfGAP with RhoGAP domain, ankyrin repeat and PH domain 2	2,633	0,004
SLC25A18	solute carrier family 25, member 18	2,107	0,005
GPRC5B	G protein-coupled receptor, class C, group 5, member B	3,417	0,005
LRIG1	leucine rich repeats and immunoglobulin like domains 1	2,598	0,005
HEPACAM	hepatic and glial cell adhesion molecule	3,055	0,005
KCNJ16	potassium channel, inwardly rectifying subfamily J, member 16	2,827	0,007
ADGRG1	adhesion G protein-coupled receptor G1	3,652	0,008
TPD52L1	tumor protein D52-like 1	3,906	0,008
SLC35B2	solute carrier family 35, member B2	2,149	0,009
CA1	carbonic anhydrase I	2,364	0,009
SLC2A5	solute carrier family 2, member 5	2,787	0,009
C17orf62	chromosome 17 open reading frame 62	2,708	0,009
MAOB	monoamine oxidase B	2,321	0,011
ADCY2	adenylate cyclase 2	2,626	0,011
GPR75	G protein-coupled receptor 75	2,438	0,011
CHD9	chromodomain helicase DNA binding protein 9	2,304	0,011
PHYHD1	phytanoyl-CoA dioxygenase domain containing 1	2,151	0,011
FCGR3B	Fc fragment of IgG, low affinity IIIb	3,156	0,011
ZGRF1	zinc finger, GRF-type containing 1	2,339	0,011
RGS12	regulator of G-protein signaling 12	2,540	0,012
CYP2R1	cytochrome P450, family 2, subfamily R, polypeptide 1	2,562	0,012
NPL	N-acetylneuraminate pyruvate lyase	3,879	0,012
RARRES3	retinoic acid receptor responder 3	3,551	0,014
HHATL	hedgehog acyltransferase-like	2,416	0,014
LGI4	leucine-rich repeat LGI family, member 4	3,816	0,014
TRIM9	tripartite motif containing 9	2,351	0,015
HTRA1	HtrA serine peptidase 1	3,371	0,015
AK1	adenylate kinase 1	2,217	0,015
P3H2	prolyl 3-hydroxylase 2	2,798	0,015

SPARCL1	SPARC like 1	2,064	0,015
IGSF11	immunoglobulin superfamily, member 11	2,307	0,016
GRIA4	glutamate receptor, ionotropic, AMPA 4	4,702	0,016
PCBP4	poly(rC) binding protein 4	2,061	0,016
RABGEF1	RAB guanine nucleotide exchange factor 1	2,206	0,017
SORL1	sortilin-related receptor, L(DLR class) A repeats containing	2,484	0,017
GIMAP7	GTPase, IMAP family member 7	2,343	0,017
ATF6B	activating transcription factor 6 beta	2,165	0,018
SERPINB9	serpin peptidase inhibitor, member 9	2,057	0,018
CAPN2	calpain 2	2,232	0,018
KATNIP	katanin interacting protein	2,665	0,019
TFRC	transferrin receptor	2,305	0,021
ITPKB	inositol-trisphosphate 3-kinase B	2,223	0,021
METTL7A	methyltransferase like 7A	4,445	0,021
RRAGC	Ras related GTP binding C	3,011	0,021
KLHL32	kelch-like family member 32	2,137	0,021
ZDHHC11B	zinc finger, DHHC-type containing 11B	4,607	0,021
CP	ceruloplasmin	3,321	0,021
LIFR	leukemia inhibitory factor receptor alpha	2,032	0,021
EFEMP1	EGF containing fibulin-like extracellular matrix protein 1	3,777	0,021
TYRO3	TYRO3 protein tyrosine kinase	2,200	0,021
ZFHX4	zinc finger homeobox 4	2,158	0,021
ART3	ADP-ribosyltransferase 3	2,441	0,022
MT1F	metallothionein 1F	3,210	0,022
NPC1	Niemann-Pick disease, type C1	3,323	0,023
KCNN3	potassium channel, calcium activated intermediate/small conductance subfamily N alpha, member 3	3,254	0,023
KCNJ10	potassium channel, inwardly rectifying subfamily J, member 10	3,252	0,023
C7orf49	chromosome 7 open reading frame 49	2,575	0,024
APLNR	apelin receptor	3,334	0,024
LGI3	leucine-rich repeat LGI family, member 3	2,881	0,024
MTUS1	microtubule associated tumor suppressor 1	2,017	0,025
TP53BP2	tumor protein p53 binding protein 2	3,183	0,026
IGFBP7	insulin like growth factor binding protein 7	4,001	0,026
PFKFB3	6-phosphofructo-2-kinase/fructose-2,6-biphosphatase 3	3,684	0,028
LRIG1	leucine rich repeats and immunoglobulin like domains 1	2,982	0,028
RPS6KA1	ribosomal protein S6 kinase, polypeptide 1	2,341	0,029
EFHD1	EF-hand domain family member D1	2,539	0,029
MYCT1	myc target 1	2,114	0,031
OPTN	optineurin	2,061	0,031
NDUFAF6	NADH dehydrogenase (ubiquinone) complex I, assembly factor 6	2,153	0,031
ALG6	ALG6, alpha-1,3-glucosyltransferase	2,552	0,031
SLCO2B1	solute carrier organic anion transporter family, member 2B1	3,505	0,031
DPYSL2	dihydropyrimidinase-like 2	2,120	0,031
BMP2K	BMP2 inducible kinase	2,439	0,031
SLC14A1	solute carrier family 14, member 1	2,971	0,032
GJA1	gap junction protein alpha 1	2,902	0,032
STX16-NPEPL1	STX16-NPEPL1 readthrough (NMD candidate)	2,014	0,033
TOB2	transducer of ERBB2, 2	2,099	0,033

MT1E	metallothionein 1E	2,822	0,034
CCDC88A	coiled-coil domain containing 88A	2,007	0,035
LIG4	ligase IV, DNA, ATP-dependent	2,032	0,035
FRYL	FRY like transcription coactivator	2,305	0,037
IL1RL1	interleukin 1 receptor-like 1	5,867	0,037
SCRG1	stimulator of chondrogenesis 1	2,636	0,037
MT1L	metallothionein 1L	2,136	0,038
PROX1	prospero homeobox 1	3,831	0,038
IL13RA1	interleukin 13 receptor, alpha 1	2,235	0,039
BAZ2B	bromodomain adjacent to zinc finger domain 2B	2,220	0,039
BRICD5	BRICHOS domain containing 5	2,190	0,039
CLASP2	cytoplasmic linker associated protein 2	2,201	0,041
GRID2	glutamate receptor, ionotropic, delta 2	2,545	0,041
ARAP2	ArfGAP with RhoGAP domain, ankyrin repeat and PH domain 2	2,076	0,041
STON2	stonin 2	2,567	0,042
ZDHHC9	zinc finger, DHHC-type containing 9	2,504	0,042
SUSD2	sushi domain containing 2	2,042	0,042
CSNK1E	casein kinase 1, epsilon	2,258	0,042
SERPINA3	serpin peptidase inhibitor, clade A, member 3	8,344	0,043
TRPS1	trichorhinophalangeal syndrome I	2,442	0,043
MAL	mal, T-cell differentiation protein	2,797	0,043
PTTG2	pituitary tumor-transforming 2	2,020	0,044
ADIRF	adipogenesis regulatory factor	3,469	0,044
LEAP2	liver expressed antimicrobial peptide 2	2,549	0,044
MT1C	metallothionein 1C	2,822	0,044
WFS1	Wolfram syndrome 1 (wolframin)	2,125	0,044
TMPRSS5	transmembrane protease, serine 5	2,535	0,045
MT1X	metallothionein 1X	3,766	0,045
ALMS1	Alstrom syndrome protein 1	2,117	0,045
MT1G	metallothionein 1G	2,391	0,046
MT1B	metallothionein 1B	2,624	0,046
STOM	stomatin	2,159	0,047
ZDHHC11	zinc finger, DHHC-type containing 11	2,072	0,047
MAP3K12	mitogen-activated protein kinase kinase kinase 12	2,044	0,047
EFHC2	EF-hand domain (C-terminal) containing 2	2,218	0,047
MS4A7	membrane-spanning 4-domains, subfamily A, member 7	2,194	0,047
RGCC	regulator of cell cycle	2,359	0,048
WIF1	WNT inhibitory factor 1	2,453	0,048
CHST11	carbohydrate sulfotransferase 11	2,294	0,049
PLLP	plasmolipin	2,161	0,049
CD34	CD34 molecule	2,181	0,049
HEY2	hes-related family bHLH transcription factor with YRPW motif 2	2,494	0,049

Table 4. Genes decreased in centenarians *vs* young individuals in hippocampus

Gene symbol	Gene name	Fold Change	p-value
PANK1	pantothenate kinase 1	-3,606	0,001
ZNF432	zinc finger protein 432	-2,460	0,001
FOXP1	forkhead box P1	-2,842	0,002
TP63	tumor protein p63	-2,083	0,002
F11	coagulation factor XI	-2,823	0,003
PNLDC1	poly(A)-specific ribonuclease (PARN)-like domain containing 1	-2,009	0,004
SPTLC3	serine palmitoyltransferase, long chain base subunit 3	-6,634	0,004
SUFU	SUFU negative regulator of hedgehog signaling	-2,025	0,005
CDON	cell adhesion associated, oncogene regulated	-2,343	0,005
NIPAL2	NIPA-like domain containing 2	-2,488	0,006
SLC38A6	solute carrier family 38, member 6	-2,415	0,006
CCDC36	coiled-coil domain containing 36	-2,167	0,007
SPRY3	sprouty RTK signaling antagonist 3	-2,104	0,009
SPRY3	sprouty RTK signaling antagonist 3	-2,104	0,009
CRY2	cryptochrome circadian regulator 2	-2,277	0,009
NARS	asparaginyl-tRNA synthetase	-2,337	0,009
SWI5	SWI5 homologous recombination repair protein	-2,227	0,009
TMEM246	transmembrane protein 246	-2,751	0,011
LOXHD1	lipoxygenase homology domains 1	-2,089	0,011
OSR1	odd-skipped related transciption factor 1	-2,059	0,011
SLIT2	slit guidance ligand 2	-2,304	0,011
SERPINF1	serpin peptidase inhibitor, clade F (alpha-2 antiplasmin, pigment epithelium derived factor), member 1	-3,020	0,012
GREB1	growth regulation by estrogen in breast cancer 1	-2,386	0,012
PTGDR	prostaglandin D2 receptor	-2,139	0,013
ERGIC2	ERGIC and golgi 2	-2,112	0,014
GALNT14	polypeptide N-acetylgalactosaminyltransferase 14	-2,413	0,014
EPGN	epithelial mitogen	-2,219	0,015
POGZ	pogo transposable element derived with ZNF domain	-2,156	0,015
TPMT	thiopurine S-methyltransferase	-2,952	0,015
SLC5A5	solute carrier family 5, member 5	-2,007	0,015
CRYZ	crystallin zeta	-2,136	0,0161
CTSK	cathepsin K	-2,426	0,017
MAP2K6	mitogen-activated protein kinase kinase 6	-2,292	0,018
HYOU1	hypoxia up-regulated 1	-2,281	0,021
CHRNA6	cholinergic receptor, nicotinic alpha 6	-2,333	0,021
ARSG	arylsulfatase G	-2,226	0,026
GOLGA6L22	golgin A6 family-like 22	-2,042	0,027
FKBP4	FK506 binding protein 4	-2,539	0,027
NNAT	neuronatin	-3,885	0,028
DIO2	deiodinase, iodothyronine, type II	-2,003	0,028
DNAJA1	DnaJ (Hsp40) homolog, subfamily A, member 1	-2,242	0,028
RNF17	ring finger protein 17	-2,458	0,029
UHRF2	ubiquitin-like with PHD and ring finger domains 2	-2,056	0,031
ZBTB10	zinc finger and BTB domain containing 10	-2,757	0,031
EFEMP2	EGF containing fibulin-like extracellular matrix protein 2	-2,211	0,032

HTT	huntingtin	-2,304	0,033
GPRC5A	G protein-coupled receptor, class C, group 5, member A	-2,135	0,033
LNX2	ligand of numb-protein X 2	-2,145	0,033
SSTR2	somatostatin receptor 2	-3,392	0,033
DFNA5	deafness, autosomal dominant 5	-3,103	0,035
GALK2	galactokinase 2	-2,131	0,036
PDK3	pyruvate dehydrogenase kinase, isozyme 3	-2,522	0,037
SLC22A6	solute carrier family 22 (organic anion transporter), member 6	-2,292	0,037
SLC25A30	solute carrier family 25, member 30	-2,068	0,037
ULK1	unc-51 like autophagy activating kinase 1	-2,263	0,038
LAMA1	laminin, alpha 1	-2,158	0,038
HSPH1	heat shock 105kDa/110kDa protein 1	-2,238	0,039
EXT1	exostosin glycosyltransferase 1	-2,383	0,041
UAP1	UDP-N-acetylglucosamine pyrophosphorylase 1	-2,181	0,041
CD83	CD83 molecule	-2,028	0,043
TSPAN18	tetraspanin 18	-9,200	0,043
ANGPT4	angiopoietin 4	-2,012	0,045
PPM1G	protein phosphatase, Mg2+/Mn2+ dependent, 1G	-2,233	0,046
C9orf72	chromosome 9 open reading frame 72	-2,258	0,046
GOLGA6L6	golgin A6 family-like 6	-2,060	0,046
PTGR1	prostaglandin reductase 1	-2,385	0,046
LAMB1	laminin, beta 1	-2,192	0,047
FAM134A	family with sequence similarity 134, member A	-2,903	0,047
TBPL1	TBP-like 1	-3,950	0,048
MPP6	membrane protein, palmitoylated 6	-3,956	0,048
NREP	neuronal regeneration related protein	-2,659	0,048
GCKR	glucokinase regulator	-2,102	0,049
KLF5	Kruppel-like factor 5	-2,417	0,049
ZFPM2	zinc finger protein, FOG family member 2	-3,366	0,049
